# Mitochondrial Uncoupling Protein 1 Overexpression Increases Yield in *Nicotiana tabacum* under Drought Stress by Improving Source and Sink Metabolism

**DOI:** 10.3389/fpls.2017.01836

**Published:** 2017-11-01

**Authors:** Pedro Barreto, Juliana E. C. T. Yassitepe, Zoe A. Wilson, Paulo Arruda

**Affiliations:** ^1^Centro de Biologia Molecular e Engenharia Genética, Universidade Estadual de Campinas, Campinas, Brazil; ^2^Joint Research Center for Genomics Applied to Climate Change (UMIP GenClima), Campinas, Brazil; ^3^Empresa Brasileira de Pesquisa Agropecuária, Embrapa Informática Agropecuária, Campinas, Brazil; ^4^School of Biosciences, University of Nottingham, Loughborough, United Kingdom; ^5^Departamento de Genética, Evolução e Bioagentes, Instituto de Biologia, Universidade Estadual de Campinas, Campinas, Brazil

**Keywords:** UCP1, drought stress, mitochondria, photosynthesis, yield

## Abstract

Mitochondrial uncoupling proteins (UCPs) sustain mitochondrial respiration independent of intracellular ATP concentration. Uncoupled respiration is particularly beneficial under stress conditions, during which both photosynthesis and respiration may be impaired. Sustaining carbon fixation during the reproductive phase is essential for plants to develop viable pollen grains and for seed setting. Here, we examined whether UCP1 overexpression (UCP1-oe) would help tobacco plants cope with drought stress during reproductive development. We observed that WT and UCP1-oe plants lost water at the same rate under moderate drought stress, but that UCP1-oe lines regained water faster upon rewatering. UCP1-oe plants maintained higher levels of respiration and photosynthesis and decreased H_2_O_2_ content in the leaves during the drought stress period. We examined whether UCP1-oe impacts reproductive tissues and seed production by monitoring the progress of flower development, focusing on the early stages of pollen formation. UCP1-oe lines induced the expression of mitochondrial genes and increased mtDNA content in reproductive tissues, which increased the consumption of carbohydrates and reduced H_2_O_2_ content and pollen disturbances. Finally, the beneficial impact of UCP1-oe on the source and sink organs resulted in an increased seed size and number under both control conditions and drought stress.

## Introduction

Plants face a range of adverse conditions that impose metabolic constraints during their lifecycle. Among these stresses, water deficit is perhaps the most important because it affects both cell energy supply and demand (Flexas et al., 2005; Atkin and Macherel, 2009). The consequences of drought stress are not limited to a cellular context but extend to whole plant physiology. The effect of water deficit on plant energy status is especially harmful during reproduction, where there is competition for nutrients between newly established sink tissues (flowers) and roots from source tissues (leaves) (Lemoine et al., 2013). Because of that water deficit is the major abiotic stress factor affecting crop development and yield (Lemoine et al., 2013). In the past few years, the frequency and intensity of drought have increased in many regions around the globe, and there are predictions of further increases as the result of global climate change (IPCC, 2014).

Drought stress inhibits photosynthesis, causing reduced production of photoassimilates (Flexas et al., 2006; Atkin and Macherel, 2009; Osakabe et al., 2014). During drought stress, leaves close their stomata to prevent water loss, which in turn reduces CO_2_ intake from the atmosphere and consequently affects carbon assimilation (Atkin and Macherel, 2009; Osakabe et al., 2014). Most of the carbon fixed in mature autotrophic leaves (source tissues) is used to synthesize sucrose for loading into phloem to be exported to roots and reproductive tissues (sink tissues; Lemoine et al., 2013). Sucrose loading is essential for reproductive tissue development, where flowers become the major sink of sugars to sustain the energy requirements for pollen production and initial seed setting (Wardlaw, 1990; Lemoine et al., 2013). During flower formation, mitochondrial biogenesis increases twenty fold in the pollen mother cells and the tapetum layer compared to that of vegetative tissues (Lee and Warmke, 1979; Giegé et al., 2005). The increased mitochondrial activity makes these tissues more prone to reactive oxygen species (ROS) production under stress because the electron leakage to form ROS at the ETC increases at higher membrane potentials (Müller and Rieu, 2016).

Because mitochondria and chloroplasts are highly interconnected organelles, the maintenance of mitochondrial respiration would help to alleviate the intracellular effects of water limitation (Atkin and Macherel, 2009). Under drought stress, while carbon assimilation is impaired, plants continue to receive light and produce reducing power in the form of NADPH at the photosystems (Osakabe et al., 2014). The excess reducing power accumulated in the chloroplasts might be exported and oxidized inside the mitochondria, which would alleviate photooxidation of chloroplast components and reduce the impairment of the Calvin cycle (Noguchi and Yoshida, 2008). Photorespiration would also be improved because it is limited by the oxidation of glycine to serine in the mitochondria in a reaction dependent on NAD+ regeneration by the mitochondrial electron transport chain (Sweetlove et al., 2006; Atkin and Macherel, 2009; Timm et al., 2012). The problem with this equation is that mitochondrial respiration in mature leaves is also inhibited during drought stress (Ribas-Carbo et al., 2005; Galmes et al., 2007; Atkin and Macherel, 2009; Dahal et al., 2014). One of the hypotheses relies on the fact that most of the ATP demands in mature leaves are used for sucrose synthesis and phloem loading; therefore, intracellular ATP content may increase during drought and restrict mitochondrial respiration adenylate (Atkin and Macherel, 2009). It is also possible that mitochondrial respiration decreases because of a limitation on substrates provided by photosynthesis or a reduction in activity of mitochondrial enzymes (Flexas et al., 2005).

Nevertheless, plant mitochondria possess mechanisms to sustain respiration independently of adenylate control. The mitochondrial uncoupling proteins (Vercesi et al., 2006) (UCPs) and alternative oxidases (Vanlerberge and McIntosh, 1997) (AOXs) sustain mitochondrial respiration that is uncoupled from ATP synthesis. The overexpression of AOX protects tobacco plants from drought stress by maintaining mitochondrial respiration (Dahal et al., 2014, 2015). In durum wheat, a drought-tolerant cereal plant, mitochondria exhibit high UCP activity, suggesting this protein may be part of the tolerance mechanism (Pastore et al., 2007). Overexpression of UCP1 (UCP1-oe) protects plants from abiotic (Brandalise et al., 2003; Smith et al., 2004; Begcy et al., 2011; Chen et al., 2013; Barreto et al., 2014) and biotic stresses (Chen et al., 2013). Increased stress tolerance is associated with the maintenance of mitochondrial biogenesis, respiration (Barreto et al., 2014), and carbon assimilation (Begcy et al., 2011). In contrast, knockouts of UCP1 in *Arabidopsis thaliana* decrease carbon assimilation due to impaired photorespiration (Sweetlove et al., 2006). UCP1-oe also alters the expression of chloroplast genes and induces the expression of a large subset of stress-responsive genes, which may explain the broad abiotic and biotic stress tolerance (Barreto et al., 2016).

In this work, we combined physiological assessments with gene expression and metabolomics analyses to evaluate the impact of UCP1 overexpression in flower development and seed production during control and water-limiting conditions. The data suggest that UCP1-oe increases respiration and photosynthesis in leaves under control conditions and maintains these processes during drought, which allows maintenance of normal flower metabolism and results in increased yields. The results are discussed in the context of the role of mitochondria in the coordination of source/sink metabolism as a prerequisite for sustained yield.

## Materials and methods

### Plant material and growth conditions

Tobacco plants (*Nicotiana tabacum*, ecotype SR1) (WT) and three transgenic lines (P07, P30, and P49) overexpressing *A. thaliana* UCP1 (Brandalise et al., 2003) were used in this study. These lines have been previously used by our group (Barreto et al., 2014, 2016) and others (Begcy et al., 2011). Seeds were sown in trays containing a soil:vermiculite (1:1) mixture; 15 days after planting, the seeds were transferred to 0.2-L pots. When plants reached the 5-leaf stage, they were transferred to 5-L pots and grown to maturity. Plants were grown in the greenhouse with a photoperiod of 16 h of light and 8 h of dark at 25–28°C at 400 μmol m^−2^ s^−1^.

The experimental design consisted of three conditions (control, drought, and recovery), each composed of 20 plants for a total five biological replicates for each genotype (Supplementary Figure 1A). The drought stress treatment consisted of withholding irrigation for 15 days at the beginning of the reproductive stage, which was defined as the moment the first inflorescence became visible (*T* = 0) (Supplementary Figure 1B). Leaves and flower buds were sampled at day zero (*T* = 0) and 15 days after drought stress imposition (*T* = 15). At *T* = 15, plants were again irrigated and visually monitored for recovery, and leaf and flower bud samples were collected 24 h after rewatering on day 16 (*T* = 16). Flower buds and the youngest expanded leaf were sampled for all plants, frozen in liquid nitrogen and stored at −80°C. We defined the first expanded leaf as the younger mature leaf that was fully developed before the beginning of stress. As large variations are expected when plants develop outside the growth chamber, the whole experiment was repeated twice under the same conditions. This additional experiment was designed to confirm the results by using important proxies, such as physiological parameters, RWC, and seed yield. All the data presented in this manuscript were obtained from a single trial.

### Water status

The relative water content (RWC) was determined for evaluating plant water status (Barrs and Weatherley, 1962). For each plant, twenty leaf discs (0.5 cm diameter) were sampled from the youngest expanded leaf. Leaf discs were weighed to determine the leaf fresh weight (FW). The turgid weight (TW) was determined by floating the leaf discs on water for 4 h, after which they were surface-dried and weighed. The dry weight (DW) was determined after drying the leaf discs at 80°C for 48 h. The percentage of leaf RWC was calculated as [(FW – DW)/(TW – DW)] × 100.

### Hydrogen peroxide content

Hydrogen peroxide (H_2_O_2_) content was measured in flower buds and leaves using the potassium iodide (KI) method (Junglee et al., 2014). Approximately 100 mg of tissue was collected, frozen in liquid nitrogen and ground into a fine powder. The powder was homogenized with 0.5 ml of a solution containing 0.25 ml of 0.1% w:v trichloroacetic acid (TCA), 0.5 ml 1 M KI and 0.25 ml of potassium phosphate buffer (10 mM, pH 5.8) at 4°C for 10 min. The homogenate was centrifuged at 12,000 × g for 15 min at 4°C, and 200 μl of the supernatant was transferred to UV microplate wells and incubated at room temperature for 20 min, after which the optical density (OD) measured at 350 nm. Samples and blanks were analyzed in triplicate. A calibration curve was obtained using H_2_O_2_ standard solutions prepared in 0.1% TCA. A pool of flower buds (8–12 mm in length) and the youngest expanded leaves from WT and UCP1-oe plants grown under control, drought and recovery conditions were evaluated. Five biological replicates were analyzed for each genotype and condition.

### Gas exchange and chlorophyll fluorescence measurements

Gas exchange was measured in the youngest completely expanded leaf using an infrared gas analyzer (IRGA–LCpro+, ADC Bioscientific, UK) at growth light intensity (400 μmol m^−2^ s^−1^). Stomatal conductance (G_S_), net photosynthetic rate (A_N_), respiration in the light (R_L_), and respiration in the dark (R_D_) were measured for each plant/treatment. R_L_ was estimated by the Kok method as described (Kok, 1948; Dahal et al., 2014). A_N_ was evaluated in a range varying from 0 to 120 μmol m^−2^ s^−1^ “PPFD” (increases of 10 μmol m^−2^ s^−1^ PPFD at each point). The measurements resulted in a Kok break point at 20 μmol m^−2^ s^−1^ PPFD. R_D_ was evaluated after 3 h of acclimation in the dark. A photosynthetic CO_2_ response curve was estimated by measuring A_N_ at saturating irradiance (1,500 μmol m^−2^ s^−1^ PPFD) during which CO_2_ was supplied at a range of concentrations (50, 100, 150, 200, 300, 400, 500, 750, and 1,000 μmol mol^−1^). The G_S_ values obtained from this curve were used to calculate the G_S_ inhibition by CO_2_ by comparing the values obtained at 400 and 1,000 μmol CO_2_ mol^−1^. We estimated the maximal velocity of Rubisco carboxylation (V_cmax_) as the slope of the linear phase of the CO_2_ response curve (Farquhar et al., 1980). The linear phase was defined as the maximum range where the *R*^2^ was above 0.95 (0–300 μmol mol^−1^ CO_2_).

Chlorophyll fluorescence was measured using a photosynthetic yield analyzer (MINI-PAM-II, Walz, GE). When necessary, a dark-adaptation period of 40 min was imposed. Initial (minimum) PS_II_ fluorescence in the dark-adapted state (F_0_), maximum PS_II_ fluorescence in the dark-adapted state (F_m_), maximum PS_II_ fluorescence in the light-adapted state (${\text{F}}_{{\text{m}}^{\prime }}$), steady-state fluorescence (F_s_), and initial (minimum) PS_II_ fluorescence in the light-adapted state (${\text{F}}_{0^{\prime }}$) were determined as described previously (Maxwell and Johnson, 2000). The maximal quantum yield of PS_II_ (F_v_/F_m_) was estimated as [(F_m_ – F_0_)/F_m_] and the effective PS_II_ quantum yield during illumination Y_II_ as ΔF/${\text{F}}_{{\text{m}}^{\prime }}$. Nonphotochemical energy quenching (NPQ), which is a measure of heat dissipation of absorbed light energy, was calculated as NPQ = (F_m_ – ${\text{F}}_{{\text{m}}^{\prime }}$)/${\text{F}}_{{\text{m}}^{\prime }}$. The photochemical energy quenching (_q_P) was estimated as (${\text{F}}_{{\text{m}}^{\prime }}$ – F_s_)/(${\text{F}}_{{\text{m}}^{\prime }}$ – ${\text{F}}_{0^{\prime }}$) and used to determine the ratio of reduced PS_II_ reaction centers (1 – _q_P).

### Metabolic profiling

Metabolites were isolated from flower buds as described (Barreto et al., 2016), with minor modifications. A total of 100 mg of frozen tissue was powdered in liquid nitrogen, incubated with 1 ml of extraction solution containing a chloroform:methanol:water solution (2:4:1) for 30 min on ice, and vigorously homogenized for an additional 15 min. After the addition of 1 ml of water, samples were centrifuged at 12,200 × g for 5 min for phase separation. The methanol:water phase was collected and vacuum-dried for 8 h. Metabolites were analyzed using a 1H-nuclear magnetic resonance (NMR) spectrometer as described (Barreto et al., 2016). Spectral phase and baseline corrections and the identification and quantification of the metabolites were performed using Chenomx NMR Suite 7.6 software. Five biological replicates for each genotype under control, drought, and recovery conditions were evaluated.

### DAPI staining of pollen grains and TUNEL assay

Flower buds were collected and fixed in a 3:1 ethanol:acetic acid solution. Anthers were removed from flower buds, placed on a glass slide and gently crushed to release pollen grains. Thirty microliters of DAPI staining solution (0.1 M sodium phosphate; 1 mM EDTA; 0.1% Triton X-100; 0.4 μg ml^−1^ DAPI; pH 7.0) was applied to each anther. Slides were kept in the dark for 3 h before visualization in a fluorescence microscope (361 nm absorption, 461 nm emission). Three biological replicates for each genotype were analyzed for each flower bud size.

For the TUNEL assay, fixed anthers were removed from the flower buds and embedded in paraffin as described previously (Vizcay-Barrena and Wilson, 2006). Anthers were washed with PBS (pH 7.2) and dehydrated using a graded ethanol series (30, 50, 70, 90, and 100%) at room temperature. The samples were then cleared by washing with ethanol/histoclear (2:1, 1:1, and 1:2 v/v) for 1 h each and then three times in 100% histoclear for 30 min each at room temperature. Tissues were embedded in paraffin for sectioning. Ten micrometer sections were cut using a Microm HM315 microtome attached to polylysine-coated slides, deparaffinized with histoclear, and hydrated in a graded ethanol series (20% increments). *In situ* nick end labeling of nuclear DNA fragmentation was performed in a humidity chamber for 1 h in the dark at 37°C with an *In Situ* Cell Death Detection Kit (Roche, CH) according to the manufacturer's instructions. For each experiment, a positive control was prepared by treating the sections with 1 U μl^−1^ DNase I for 10 min at 37°C before labeling as above. The negative controls were labeled in parallel, except for the absence of the enzyme terminal deoxynucleotidyl transferase (TdT). The reaction solution was removed by rinsing the slides 3 times with PBS (pH 7.2). Samples were analyzed under a fluorescence confocal scanning microscope (Leica TCS SP2 Confocal). The fluorescence filter was set to view the green fluorescence of fluorescein at 520 nm.

### Gene expression and mtDNA quantification

Total RNA was isolated from flower buds using a Spectrum Plant Total RNA Kit (Sigma-Aldrich). The first-strand cDNA was synthesized using a RevertAid First Strand cDNA Synthesis kit (Fermentas, US) according to the manufacturer's protocol. Mitochondrial DNA was quantified from total DNA isolated from flower buds using the Plant DNAzol reagent (Invitrogen, US). Real-time PCR was performed using the ABI PRISM 7500 system (Applied Biosystems, US) with SYBR Green dye (Applied Biosystems, US). At least triplicate reactions were performed with four biological replicates, and the results were expressed relative to the expression level of the *N. tabacum PP2A* gene in each sample using the 2^−ΔΔCT^ method. Some of the primers were designed for *Nicotiana sylvestris* because reference data for *N. tabacum* were unavailable in the NCBI database (Supplementary Table 1). The PCR products for primers designed for *N. sylvestris* were sequenced after amplification of *N. tabacum* for validation. All values are presented as fold changes of transgenic lines relative to those of WT. Student's *t*-test was performed to determine significant changes (^*^P < 0.1, ^**^P < 0.05, ^***^P < 0.01).

### Western blot

Total soluble proteins were isolated from WT and transgenic lines by grinding frozen flower tissue in protein extraction buffer (50 mM Tris, pH 8.5; 1 mM EDTA; 1 mM DTT; 5 mM PMSF; 0.2% Triton X-100; 5 mM benzamidine; 5% w/v PVPP). A cOmplete™, EDTA-free Protease Inhibitor Cocktail (Roche, SW) was added to the protein extraction buffer before use. The proteins were separated on 12% SDS-PAGE gels, transferred to nylon membranes, and blotted using UCP1 (1:3,000) and Actin11 (1:1,500) polyclonal antibodies raised against Arabidopsis (Agrisera, SE). Bands were detected using an ImageQuant LAS500 (GE Healthcare, UK) system with the SuperSignal West Pico chemiluminescent substrate (Thermo-Scientific, US).

### Yield and seed size quantification

Five flower buds from each plant were tagged at the end of the drought stress treatment. The tagged buds were kept for seed set, the mature pods were collected at the same time for all genotypes, and their entire seed content weighed. The yield was estimated as the mass of seeds per pod. For seed size analysis, an image field containing ~100 seeds was imaged, and seed area was measured using ImageJ software. Six siliques were analyzed for each plant from five biological replicates. Approximately 2,500 seeds were imaged to estimate the average seed size for each condition.

### Statistical analysis

ANOVA with Tukey-Kramer test was used to assess the significance of changes in this research. The analyses were conducted using PRISM 6.0 (GraphPad Software Inc., US). The only exception was for principal component (PCA) and correlation analysis, on which we used the Metaboanalyst webportal (Xia et al., 2012). The metabolite and physiological data were normalized by median centering before running the analysis. The Pearson correlation coefficient was used for correlation analysis.

## Results

### UCP1-oe accelerates recovery upon rehydration after drought stress

**Figure 1:**
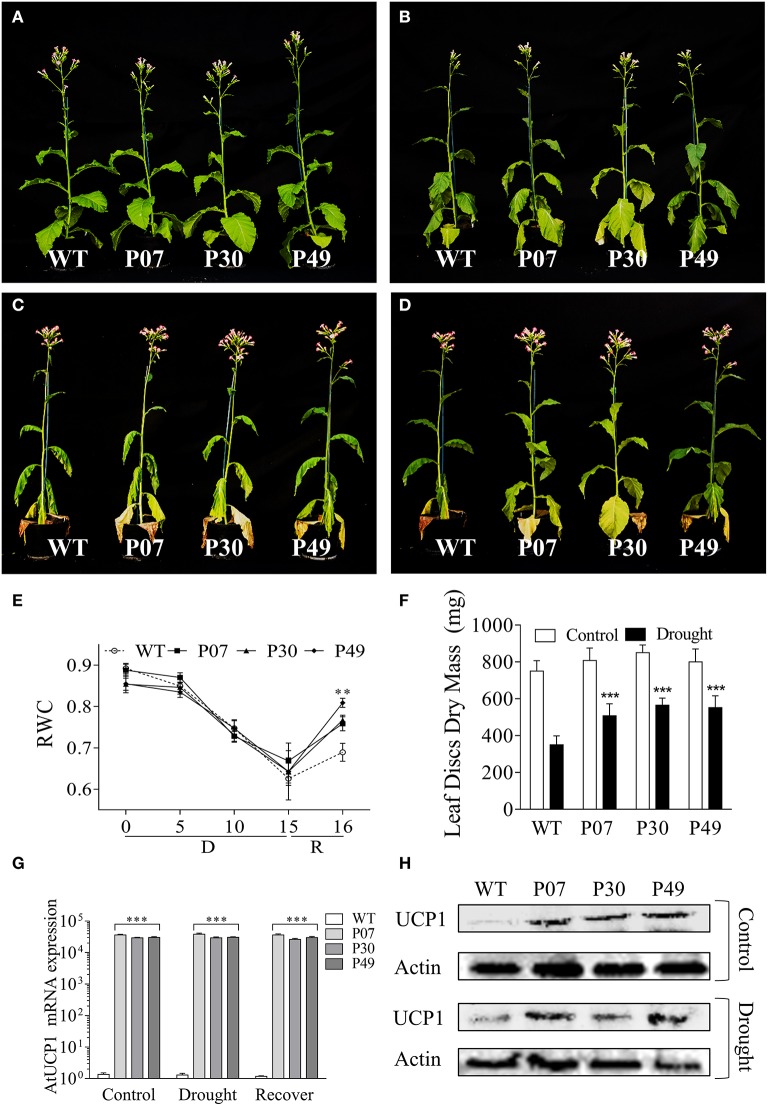
UCP1-oe lines recover faster from drought stress than do WT upon rewatering. Plants were grown until **(A)** the reproductive phase of development, after which irrigation was stopped. Plants started to show symptoms of drought stress after **(B)** 9 days without irrigation, and they were maintained without irrigation for **(C)** 15 days after the start of the drought treatment. **(D)** Transgenic plants recovered faster after 1 day of recovery. **(E)** Relative water content (RWC) was monitored on the first expanded leaf during the whole course of the experiment. **(F)** The absolute dry mass of the first fully expanded leaf (20 leaf discs) was compared between control and drought-stressed plants. **(G)** The heterologous UCP1 was overexpressed in flower buds of all three transgenic lines (P07, P30, and P49) as previously described for the leaves. **(H)** Immunoblot analysis of the flower buds of WT and transgenic lines P07, P30, and P49 under control and drought conditions was performed using an anti-UCP1 antibody. The gel loading control was estimated using an anti-β-actin antibody. Bars and points represent the means ± SD. Bars with ^***^ differ significantly from the WT (*P* < 0.01). Points with ^**^ mean that all transgenic lines differ from the WT (*P* < 0.05). D, Drought; R, Recovery.

WT and three independent UCP1-oe lines (P07, P30, and P49) were grown until the beginning of the flowering stage (Figure 1A), when the irrigation was withdrawn (IW). Plants started to show symptoms of water deficit after 9 days of IW (Figure 1B) and showed severe wilting after 15 days of IW (Figure 1C). After 15 days of IW, plants were rewatered; 24 h after irrigation resumption, it was evident that UCP1-oe lines rehydrated faster than the WT plants based on the plant turgidity (Figure 1D) and RWC (Figure 1E). The RWC at 15 days of IW decreased to 65%, which is considered moderate drought stress (Dahal et al., 2014, 2015). The decrease in RWC did not differ between WT and UCP1-oe lines. UCP1-oe lines also presented increased dry weight after the drought period compared to their non-stressed counterparts (Figure 1F). WT plants under drought stress presented 42% as much dry mass as did their well-watered counterparts, whereas the UCP1-oe lines showed 58–62% as much dry weight as did their well-watered controls. We also examined the presence of the heterologous *A. thaliana*UCP1 mRNA (Figure 1G) and UCP1 protein (Figure 1H) in flower buds. The results confirmed that heterologous UCP1 is being expressed in tobacco flower buds. Interestingly, UCP1 protein accumulation is induced by drought in flower buds of the WT.

**Figure 2:**
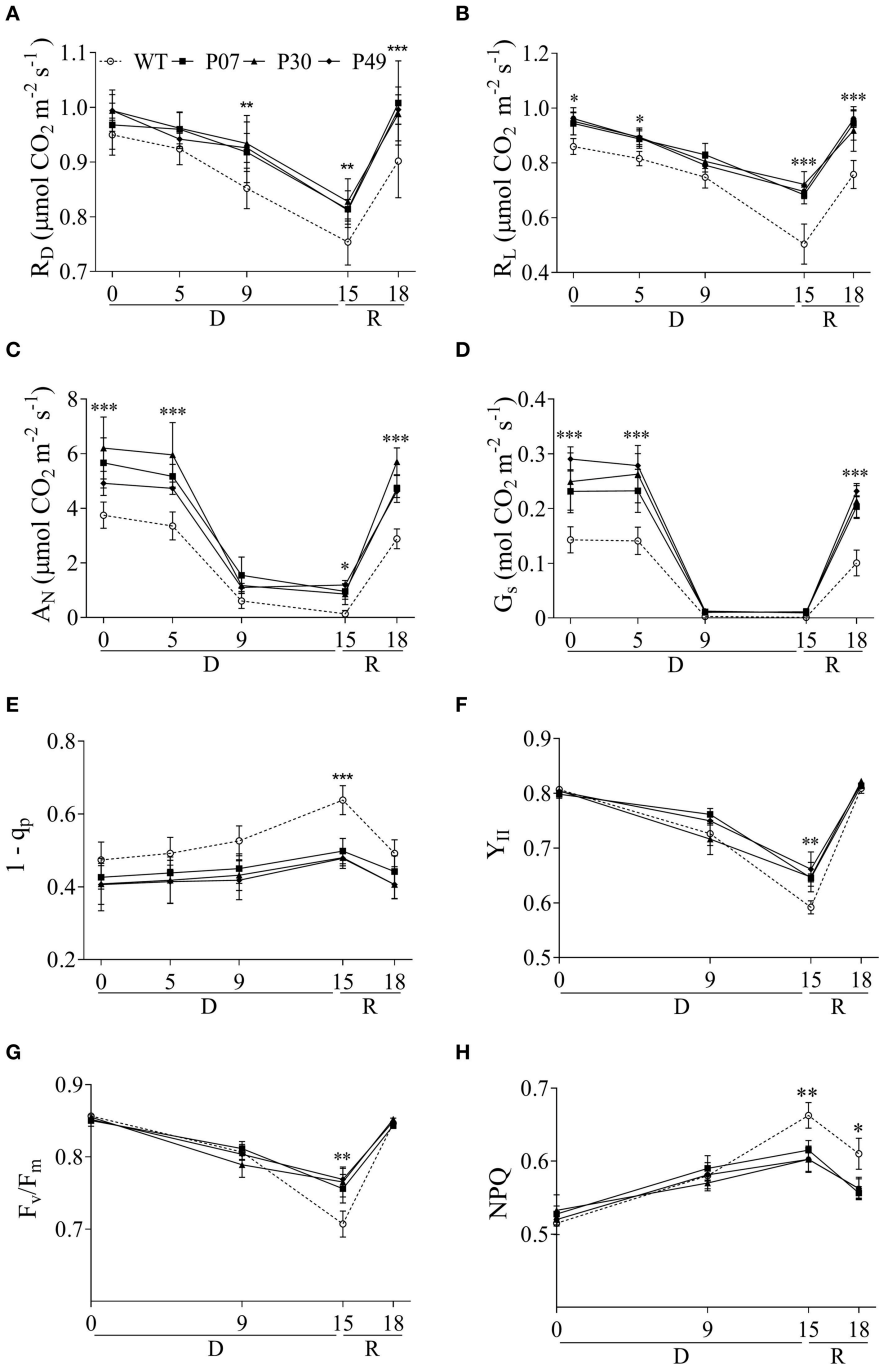
Effects of drought stress on physiological and chlorophyll fluorescence parameters of WT and *AtUCP1*-overexpressing transgenic plants. The measurements were taken at growth light intensity (400 μmol m^−2^ s^−1^) using the first expanded leaf during the whole course of the experiment. For this experiment, plants were allowed to recover from drought stress for 3 days. **(A)** Respiration in the dark: (R_D_), **(B)** respiration in the light (R_L_), **(C)** net photosynthetic rate (A_N_), **(D)** stomatal conductance (G_S_), **(E)** excitation pressure (1-_q_P), **(F)** effective PS_II_ quantum yield during illumination (Y_II_), **(G)** maximal quantum yield of PS_II_ (F_v_/F_m_), and **(H)** non-photochemical energy quenching (NPQ). Points represent the means of 5 plants ± SD. Points with ^*^, ^**^, and ^***^ mean that all three transgenic lines differ significantly from the WT (*P* < 0.1, *P* < 0.05, and *P* < 0.01, respectively). D, Drought; R, Recovery.

The increase in dry weight suggests that the UCP1-oe lines maintained physiological activities such as respiration and photosynthesis under drought stress. This agrees with the higher mitochondrial respiratory capacity of UCP1-oe (Barreto et al., 2014) and the lower respiration of UCP1 knockout tomato lines (Liu et al., 2015). Additionally, increased mitochondrial respiratory capacity is associated with enhanced tolerance to drought stress (Dahal et al., 2014, 2015). Mitochondrial respiration in the dark (R_D_) did not differ between WT and UCP1-oe lines under control conditions (Figure 2A), whereas UCP1-oe lines presented a slight but significant increase (12%) in mitochondrial respiration in the light (R_L_) (Figure 2B). Although, there is a tendency of UCP1-oe cause increased R_D_, this trend was only significant after drought treatment or during recovery (Figure 2A). In contrast, R_L_ significantly increased in UCP1-oe lines during the whole course of the experiment, but this increase was more pronounced at the end of the drought treatment (38%) and during recovery (25%) (Figure 2B).

There was an increase in net carbon assimilation (A_N_) under control conditions and water limitation as well as during the recovery period (Figure 2C). The A_N_ values after recovery increased 45% in UCP1-oe lines compared to that of WT plants. Stomatal conductance (G_S_) (Figure 2D) was also higher in UCP1-oe lines under control conditions and during recovery; nevertheless, G_S_ severely decreased under drought in a similar extent to that of WT plants. In a similar manner to the A_N_, G_S_ values after recovery were 95% higher in UCP1-oe lines as compared to the WT plants. As a larger variation is expected when growing plants in the greenhouse, we performed a second trial of this experiment where we could confirm the effect of UCP1-oe upon the physiological parameters (Supplementary Figure 2).

Chlorophyll fluorescence was used to evaluate the impact of drought stress on PS_II_. Excitation pressure (1 – _q_P) (Figure 2E), photosynthetic yield (Y_II_) (Figure 2F), the maximum photochemical efficiency of PS_II_ in the dark-adapted state (F_v_/F_m_) (Figure 2G) and nonphotochemical energy quenching (NPQ) (Figure 2H) were similar in WT and UCP1-oe throughout most of the experiment. There were only differences between WT and UCP1-oe after 15 days of IW, where Y_II_ and F_v_/F_m_ increased in UCP1-oe lines while NPQ and 1 – _q_P decreased by 10 and 23%, respectively.

**Figure 3:**
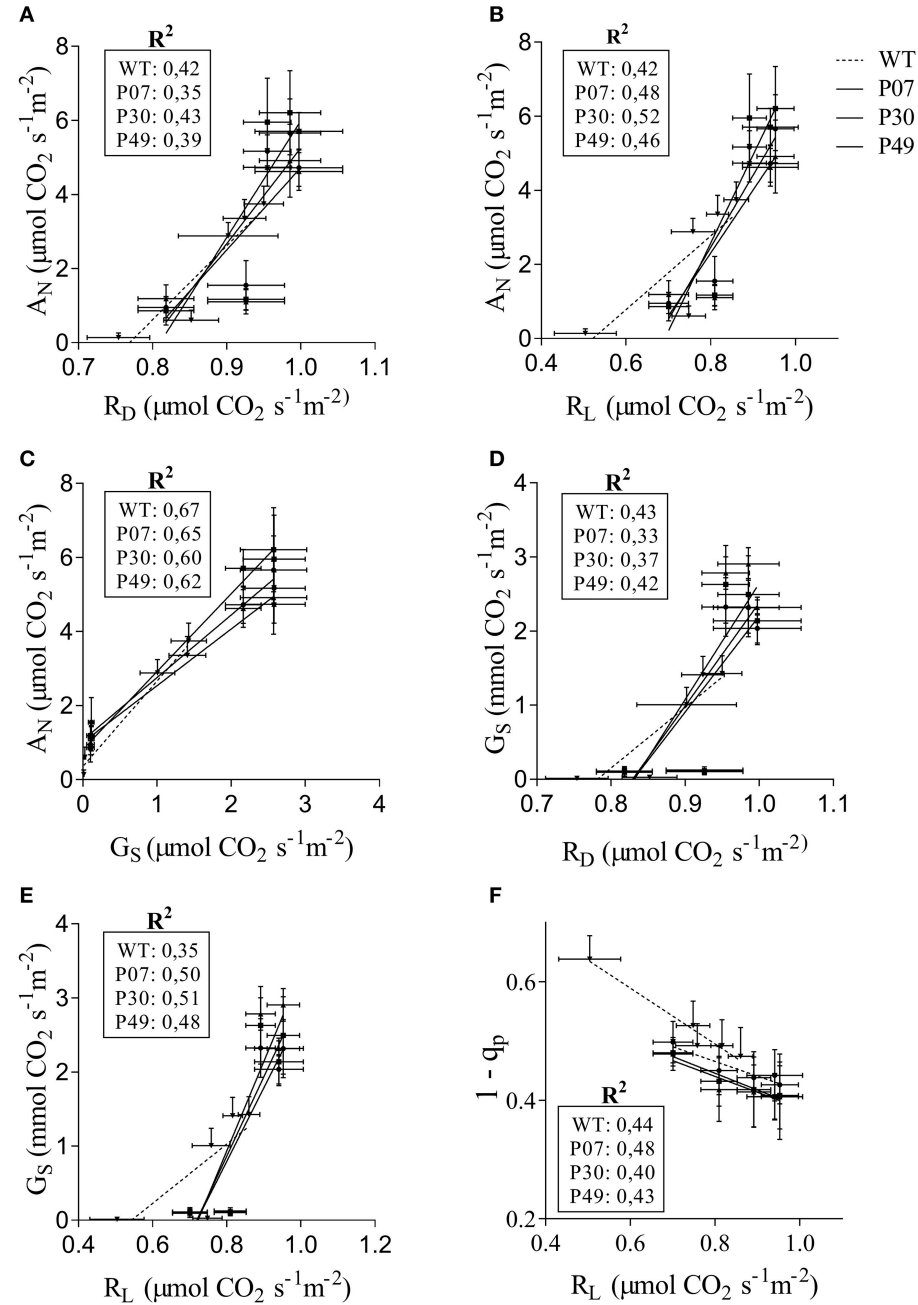
Correlation of physiological parameters in WT and transgenic lines. The relationships of **(A)** R_D_, **(B)** R_L_, and **(C)** G_S_ with A_N_ was determined for WT (dashed line) and transgenic lines (plain lines). **(D,E)** G_S_ as a function of R_D_ and R_L_, respectively. **(F)** 1-qP as a function of R_L_. The curves were calculated for each line individually. Control, drought and recovery data were pooled together for the linear regression analysis.

We next examined which of the physiological parameters evaluated present higher correlation with the observed increased in A_N_ (Figures 3A–C). When comparing the correlation between R_D_ (Figure 3A), R_L_ (Figure 3B), and G_S_ (Figure 3C) with A_N_ it is clear that G_S_ is the one with the highest correlation values in both WT and transgenic lines. Interestingly both the intercept and slope of the WT G_S_ × A_N_ correlation differ from the transgenic lines (Supplementary Table 2). This fact is also true for R_L_ × A_N_ correlation but not for R_D_ × A_N_. In the opposite direction, both the correlations R_D_ or R_L_ × G_S_ differ at intercept and slope between WT and transgenic lines (Figures 3D,E and Supplementary Table 2). As previously observed, there is a high correlation between 1 – q_p_ and R_L_ (Dahal et al., 2016) but its slope and intercept did not differ between WT and transgenic lines (Figure 3F and Supplementary Table 2).

**Figure 4:**
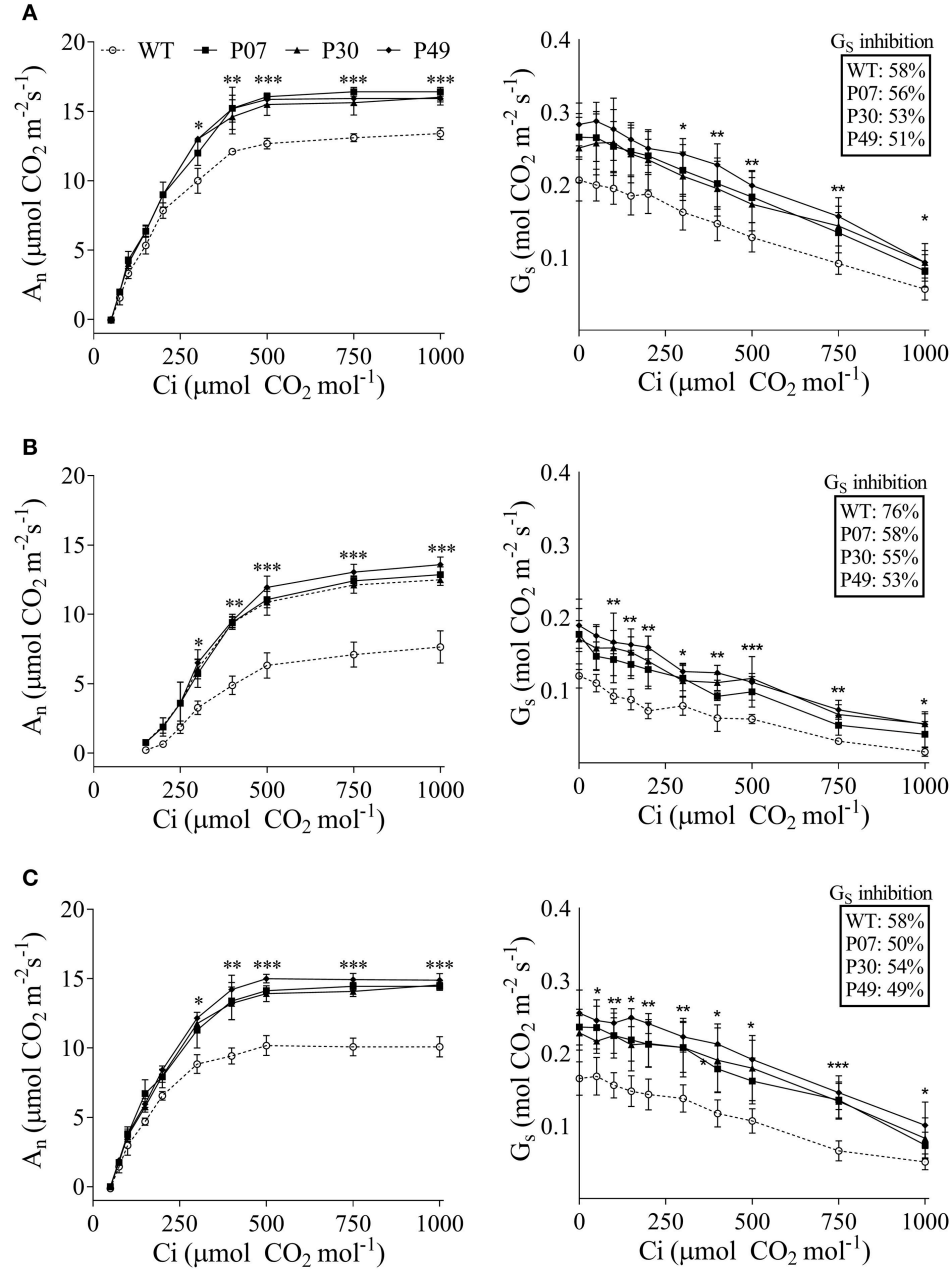
The impact of water limitation on net carbon assimilation and stomatal conductance response to CO_2_ in WT (dashed line) and transgenic plants (plain lines). The measurements were taken using the first expanded leaf during the whole course of the experiment in **(A)** control, **(B)** drought-stressed, and **(C)** recovery plants at growth light intensity. Measurements of the drought-stressed plants were taken at the end of the stress period, whereas recovery measurements were taken after 3 days of recovery under normal irrigation. Points represent the means of 5 plants ± SD. Points with ^*^, ^**^, and ^***^ mean that all three transgenic lines differ significantly from the WT (*P* < 0.1, *P* < 0.05, and *P* < 0.01, respectively).

Due to the high impact of UCP1-oe on photosynthesis, we next examined A_N_ and G_S_ as a response to varying CO_2_ concentrations (Figure 4). The slopes of the linear portion of the curves were used to estimate the maximum velocity of Rubisco carboxylation (V_cmax_) (Supplementary Table 3). There were no differences between transgenic lines and WT V_cmax_ under control and recovery conditions, while transgenic lines showed higher V_cmax_ under drought stress. In the opposite direction, A_N_ was found increased in transgenic lines at RuBP-regeneration-limited condition under control (Figure 4A), drought (Figure 4B), and recovery (Figure 4C) conditions. Differences were more pronounced in stressed plants than in control or recovery plants, where A_N_ in the WT represents 61% of that of the transgenic lines at the saturation point. As previously observed (Figure 2) the G_S_ values for UCP1-oe plants were higher as compared with WT at the entire range of CO_2_ concentration in all the conditions evaluated (Figure 4). In addition, Gs was inhibited by CO_2_ to the same extent as the UCP1-oe plants under control and recovery conditions (Figures 4A,C). On the other hand, G_S_ was less inhibited by CO_2_ in UCP1-oe during drought stress (Figure 4B).

**Figure 5:**
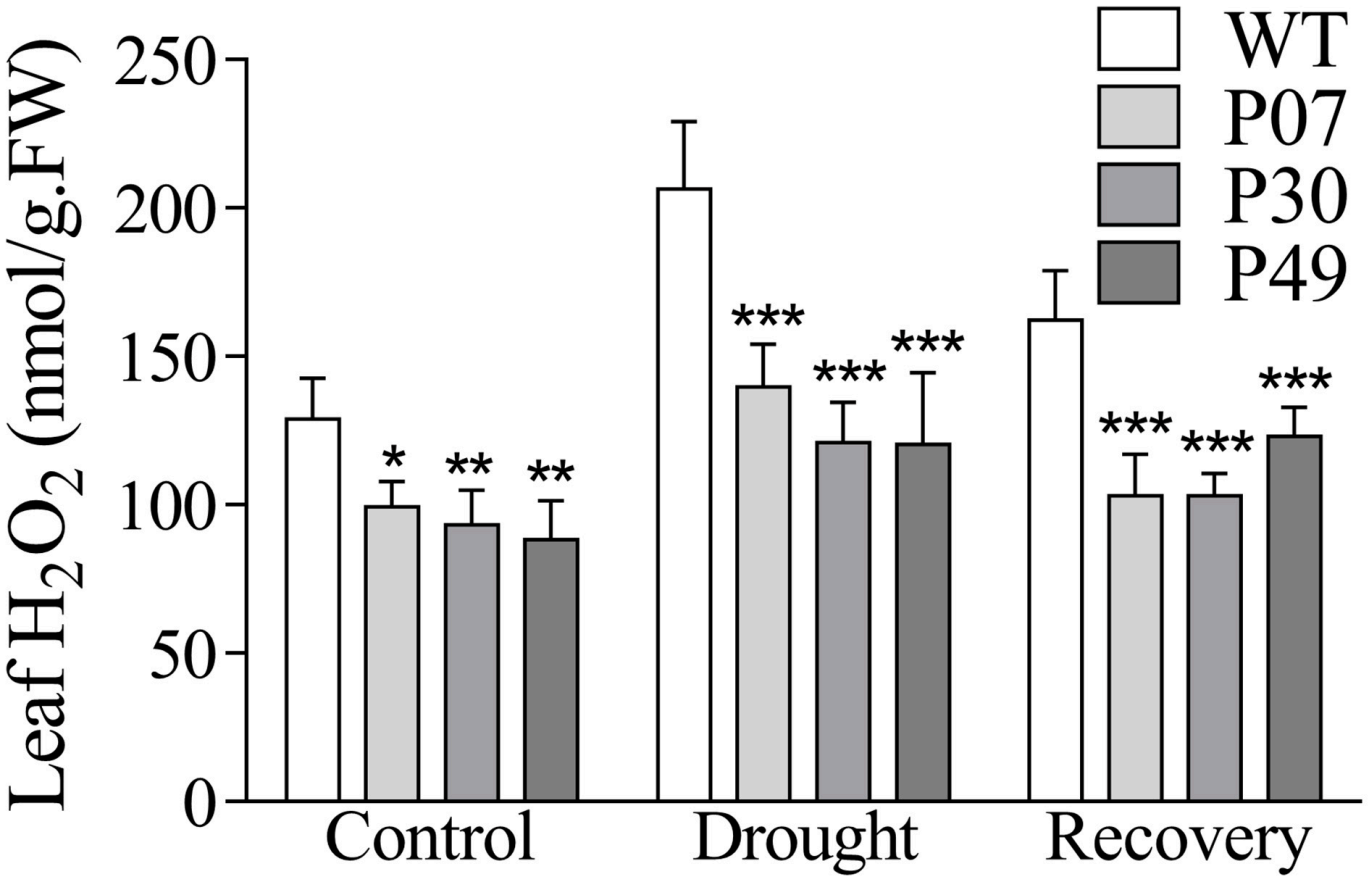
Quantification of H_2_O_2_ in leaves of WT and *AtUCP1* transgenic tobacco plants. The first expanded leaf was collected from WT and transgenic plants under control (*D* = 0), drought (*D* = 15), and recovery (*D* = 16) conditions. Bars represent the means of 5 plants ± SD. Bars with ^*^, ^**^, and ^***^ differ significantly from the WT (*P* < 0.1, *P* < 0.05, and *P* < 0.01, respectively).

The maintenance of physiological parameters during the drought might result in a less stressed cellular environment. As UCP1-oe plants have been shown to reduce H_2_O_2_ production (Smith et al., 2004; Begcy et al., 2011; Chen et al., 2013) and as the levels of H_2_O_2_ increase in response to drought (Luna et al., 2005), measuring H_2_O_2_ content represents a good proxy to infer the degree of stress imposed during the period of water withholding. As expected, UCP1-oe lines accumulated 28% less H_2_O_2_ in leaves under control, drought and recovery conditions than did WT (Figure 5). Under drought stress, WT plants showed a 60% increase in H_2_O_2_ content compared to only a 32% increase in UCP1-oe lines. During the first day of recovery, the H_2_O_2_ content in the UCP1-oe lines decreased to a level that was only 7% higher than that in the well-watered control. After the same recovery time, the WT H_2_O_2_ content increased 26% compared to that of the control.

### Impact of UCP1-oe on flower development during drought stress

**Figure 6:**
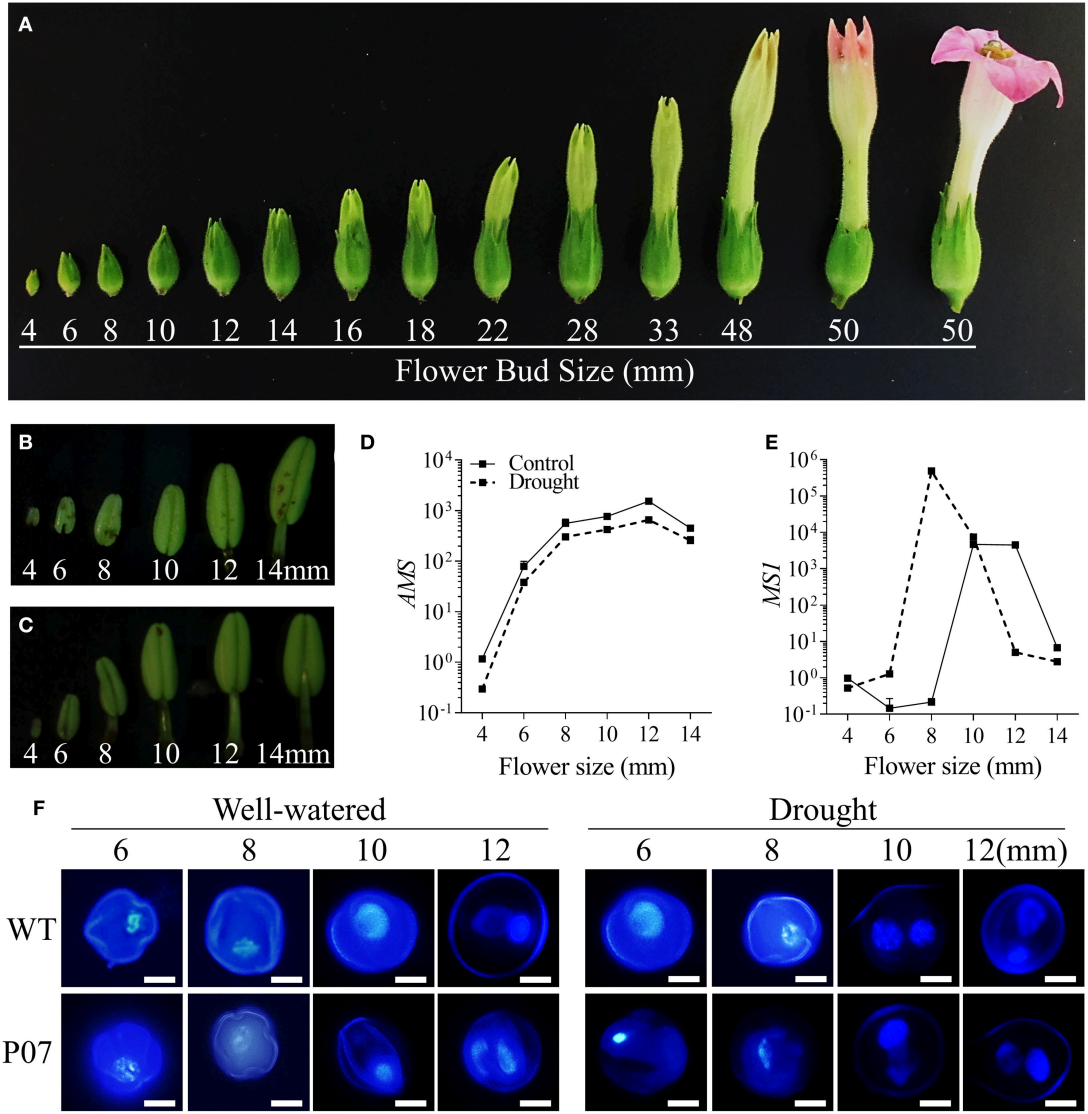
Flower and anther development in tobacco plants. **(A)** Flower development was assessed in tobacco plants based on bud size. Focusing on the early stages of pollen development, anthers was isolated from 4 to 14 mm buds from **(B)** control and **(C)** drought-stressed WT plants. The expression of **(D)** *AMS* and **(E)** *MS1* was analyzed using qRT-PCR in control and drought-stressed WT plants to identify the key initial stages in pollen development. **(F)** Pollen grains of WT and transgenic line P07 were stained with DAPI to determine correlations between bud size and pollen stage in control and drought-stressed plants. Scale bars = 10 μm.

During the reproductive stage, leaves act as source tissues to satisfy the high energy demand of flowers, especially during pollen development (Wardlaw, 1990; Lemoine et al., 2013). Therefore, it would be expected that the positive impact of UCP1-oe on photosynthesis in the leaves would be reflected in the reproductive tissues of the plants. We first characterized how flower development progresses in tobacco from early formed flower buds (4 mm) to fully developed flowers (50 mm; Figure 6A). Anthers were isolated from WT flower buds ranging from 4 to 14 mm under both control (Figure 6B) and drought stress conditions (Figure 6C). Expression profiling of the two key transcription factors (TFs) *ABORTED MICROSPORES* (*AMS*) and *MALE STERILITY 1* (*MS1*) that act during tapetum maturation (Wilson et al., 2011) was evaluated in WT plants under both control and drought stress conditions. While *AMS* expression was inhibited approximately two-fold (Figure 6D), *MS1* was induced and expressed at an earlier stage (Figure 6E) in drought-stressed plants than in their control counterparts. The faster progression of pollen development was confirmed by DAPI staining of pollen grains from WT and UCP1-oe P07 flower buds, ranging from 6 to 12 mm (Figure 6F). There were no differences in the progression of pollen development between WT and P07 plants, but it seemed that both were equally affected by drought stress. Pollen grains seemed mostly uninuclear in 10-mm flower buds in control plants, whereas most pollen grains were binuclear in plants under drought stress. Flower development was accelerated under drought stress—especially anther development, which was noticeable in anther size and stamen elongation (Figures 6B,C,F). For these reasons, we decided to further analyze 10-mm flower buds from control plants and 8-mm flower buds from drought-stressed and recovery plants. This would allow us to equalize the stages analyzed in control, drought-stressed, and recovered plants.

**Figure 7:**
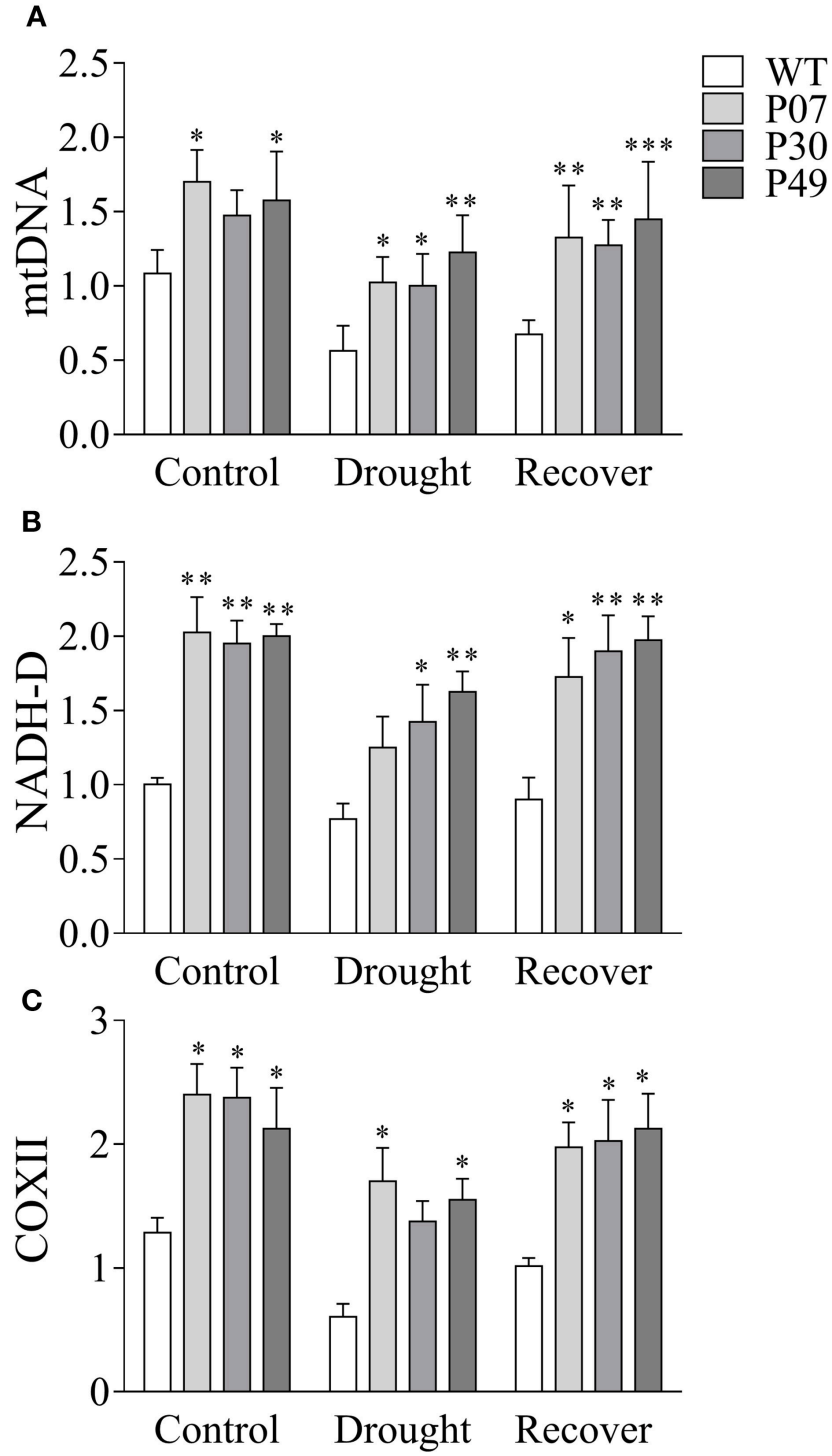
Overexpression of *AtUCP1* results in increased expression of mitochondrial transcripts in flower buds. **(A)** The mtDNA content is higher in transgenic lines in control, drought stress, and recovery conditions. The expressions of **(B)** nuclear-encoded *NADH-D* and **(C)** mtDNA-encoded *COXII* were also higher in transgenic plants in all conditions analyzed. Bars represent the means of 5 plants ± SD. Bars with ^*^, ^**^, and ^***^ differ significantly from the WT (*P* < 0.1, *P* < 0.05, and *P* < 0.01, respectively).

We first determined whether the increased mtDNA content and induction of mitochondrial gene expression that was previously shown in leaf tissues (Barreto et al., 2014) was present in flowers (Figures 6A–C). UCP1-oe lines showed a 1.6-fold increase in mtDNA content in flowers under control conditions compared to those of WT plants (Figure 7A). Under drought stress in WT plants, mtDNA content decreased to 56% of the control level, while in UCP1-oe lines it decreased to 68%. However, the mtDNA content of UCP1-oe buds was restored faster than that of their WT counterparts (76% compared to 62%) during the first 24 h of recovery. The expression of both nuclear-encoded mitochondrial *NADH-dehydrogenase* (*NADH-D*) (Figure 7B) and mitochondrial-encoded *cytochrome oxidase II* (*COXII*) (Figure 7C) was more than two-fold higher in UCP1-oe under control conditions than in WT. The expression of both genes remained elevated in UCP1-oe lines in both drought stress and recovery conditions.

**Figure 8:**
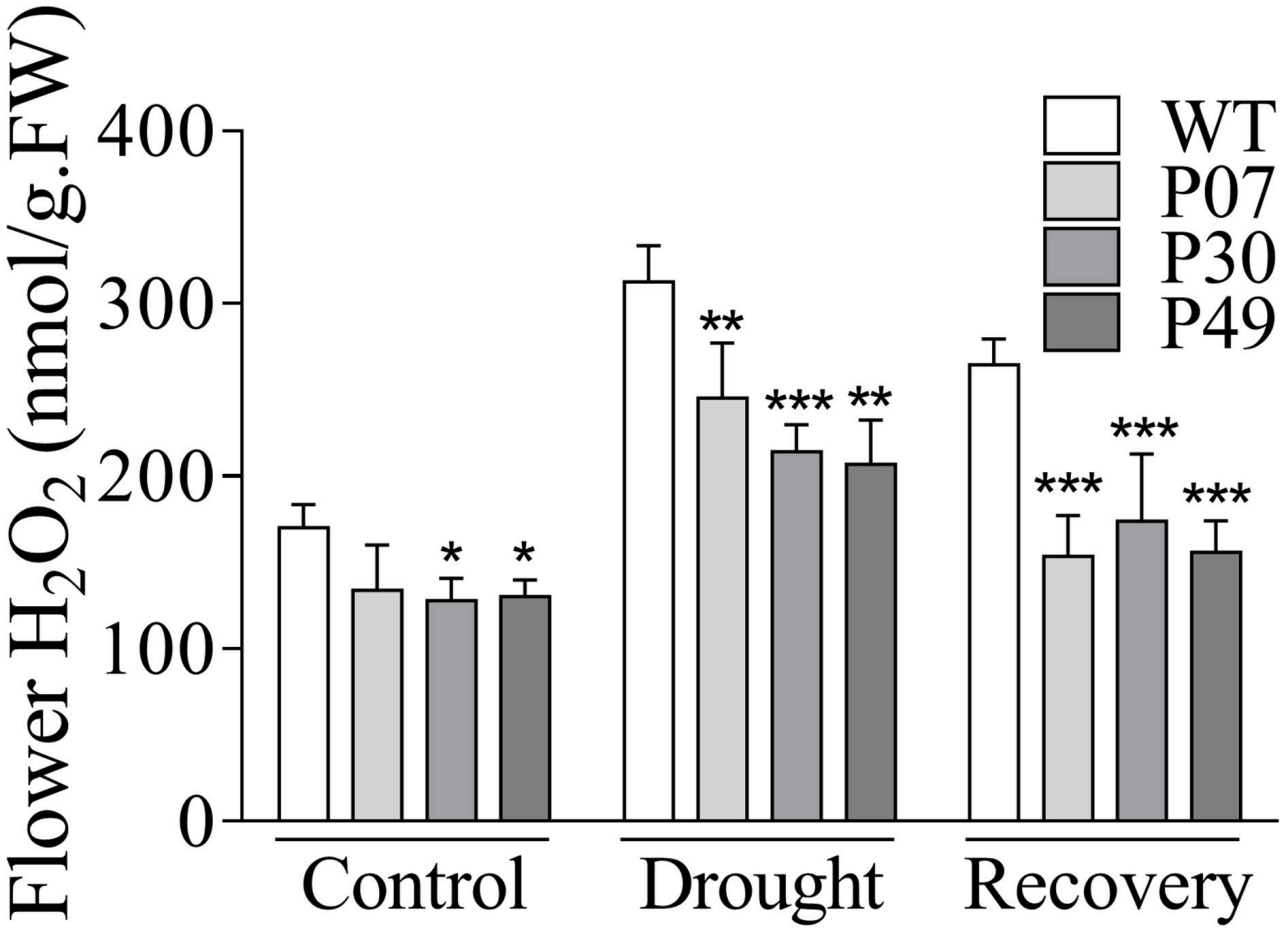
Quantification of H_2_O_2_ in flower buds of WT and *AtUCP1* transgenic tobacco plants. Flower buds (8–12 mm) were collected from WT and transgenic plants under control (*D* = 0), drought stress (*D* = 15), and recovery (*D* = 16) conditions. Bars represent the means of 5 plants ± SD. Bars with ^*^, ^**^, and ^***^ differ significantly from the WT (*P* < 0.1, *P* < 0.05, and *P* < 0.01, respectively).

Due to their higher mitochondrial content, flower tissues are much more prone to oxidative stress than are leaf tissues (Müller and Rieu, 2016). In this context, UCP1-oe might benefit flower tissues by counteracting increased ROS production. The H_2_O_2_ content in UCP1-oe flower buds corresponded to 72% of the level in those of WT (Figure 8). Under drought stress, H_2_O_2_ levels in WT plants increased by 82%, whereas in UCP1-oe, the levels increased by only 65%. As previously observed in leaves, the H_2_O_2_ content during recovery was much closer to that of the control conditions in UCP1-oe lines (123%) than in WT (155%).

### Modeling of primary metabolism and physiological response

**Figure 9:**
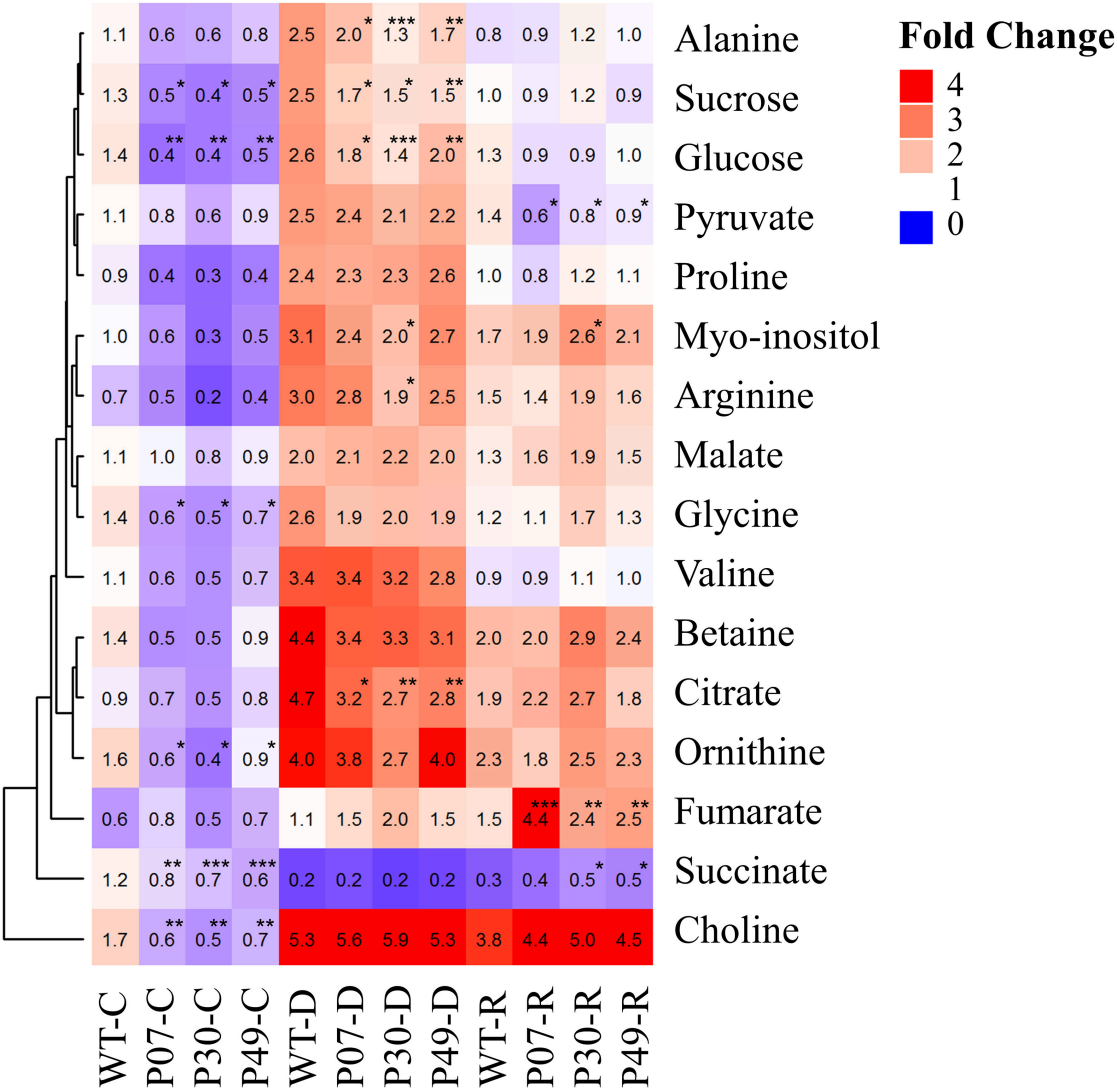
Heatmap showing the distribution of metabolites that were significantly affected by drought stress. Metabolites were isolated from WT and transgenic plants (P07, P30, and P49) under well-watered (C), drought (D), and recovery (R) conditions for metabolomics using H^1^-NMR. The average fold-change is displayed inside the enclosed cells as relative to those of WT under control conditions. Cells represent the means of 5 plants ± SD. Points with ^*^, ^**^, and ^***^ mean that the transgenic line differ significantly from the WT (*P* < 0.1, *P* < 0.05, and *P* < 0.01, respectively).

The adaptation to drought stress is often associated with the accumulation of compatible osmolytes (Kiyosue et al., 1996). We were interested in whether UCP1-oe lines would alter osmolyte profiles (Figure 9). There were no differences in the accumulation of most of the amino acids between WT and UCP1-oe under both control and drought stress conditions. Nevertheless, alanine was found to be 30% higher in WT plants under drought stress when compared to the transgenic lines. In contrast to what was expected, choline, glycine, and ornithine were slightly but significantly decreased in UCP1-oe lines under control conditions.

We also examined the possible impact on intermediates of flower energy metabolism (Figure 9). The photosynthetic assimilates sucrose and glucose were reduced in the UCP1-oe lines compared to WT under both control and drought stress conditions. Pyruvate increased in UCP1-oe lines only during recovery conditions. Intermediates of the Krebs cycle have distinct patterns of accumulation. Citrate was found to be reduced in UCP1-oe under drought stress, whereas succinate differs to that of WT under control conditions. Fumarate accumulated to high levels in UCP1-oe lines during recovery, and malate levels were unchanged in all conditions evaluated.

**Figure 10:**
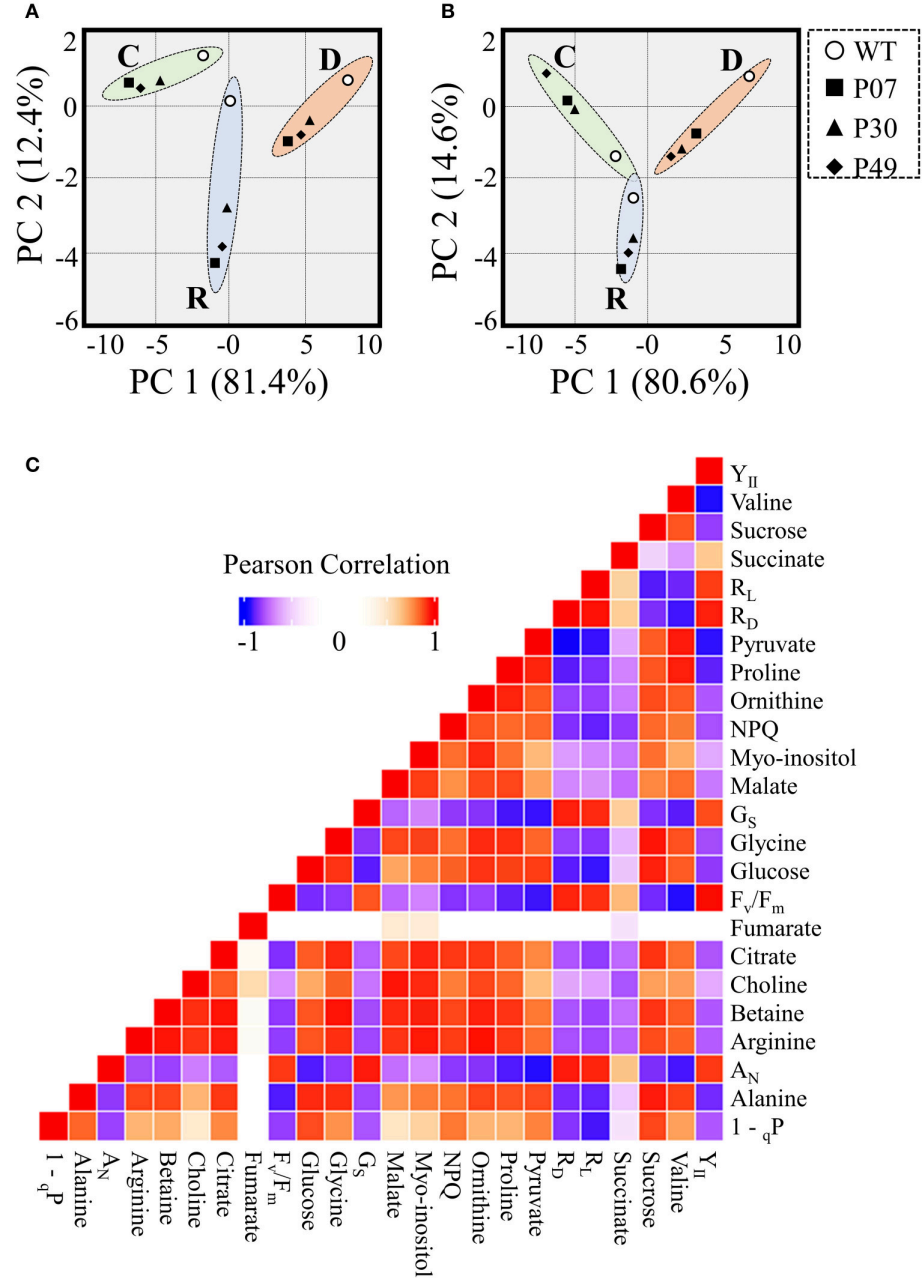
Data integration of metabolomics combined with physiological parameters of WT and transgenic lines. Principle component analysis (PCA) scores plot representation of **(A)** metabolomics or **(B)** metabolomics combined with physiological parameters from WT and transgenic lines (P07, P30, and P49). Dashed lines represent 95% confidence intervals for well-watered (C), drought (D), and recovery (R) conditions. **(C)** Pearson correlation analysis of metabolomics combined with physiological parameters.

We asked how the flower primary metabolism is linked to the physiological responses observed in WT and UCP1-oe lines exposed to drought stress (Figure 10). A principal component analysis (PCA) separate primary metabolism data into three distinct groups in accordance to the treatments (Figure 10A). When we combined primary metabolism, and physiological parameters, well-watered and recovery plants did not statistically differ from each other at 95% confidence level (Figure 10B). Also, it is important to notice that transgenic lines group together and differ from the WT across treatments. To obtain a global view of how significant alterations in primary metabolism is associated with the observed physiological response during the drought stress, we analyzed changes in correlations in WT and transgenic lines across treatments (Figure 10C). Pearson correlation coefficient values were calculated (Supplementary Table 4) for the 16 metabolites (Figure 9) and 8 physiological parameters (Figure 2). In general, metabolites correlated positively among themselves. The same pattern was observed for physiological parameters. Exceptions were found for succinate, fumarate, 1 – _q_P and NPQ. Interestingly, sugars content and succinate, which are decreased in UCP1-oe flower buds under control conditions, correlate negatively with the physiological parameters. Although, fumarate increases drastically in UCP1-oe during recovery it seems not correlate strongly with most of physiological parameters and metabolites.

### Expression profiling of flower-specific transcription factors and the TUNEL assay

**Figure 11:**
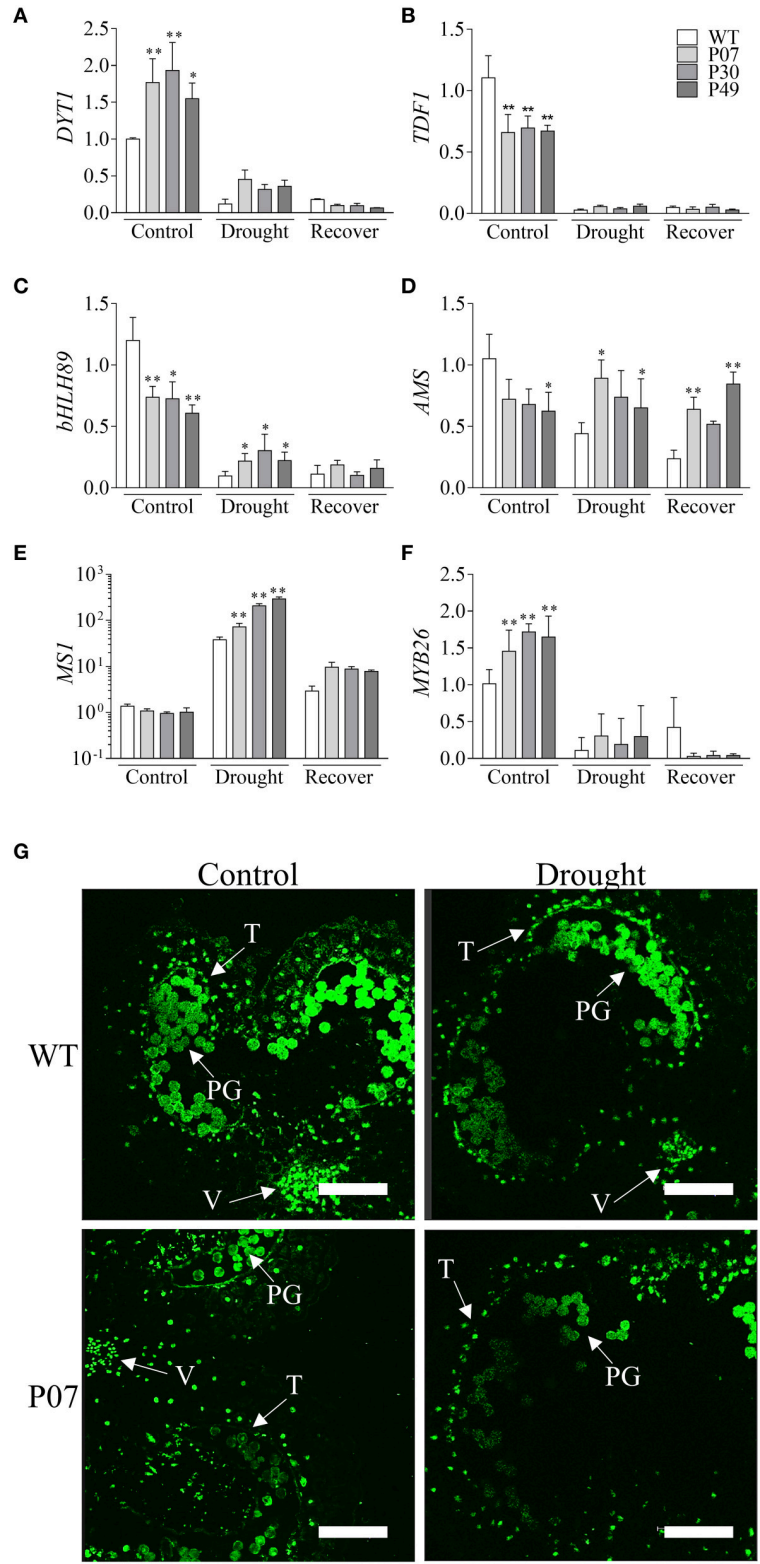
Overexpression of *AtUCP1* impacts the expression of anther-specific transcription factors but does not affect tapetum PCD during drought stress. We evaluated the expression of transcription factors that regulate the beginning of tapetum development, tapetal maturation and anther maturation. The values of **(A)** *DYT1*, **(B)** *TDF1*, **(C)** *bHLH89*, **(D)** *AMS*, **(E)** *MS1*, and **(F)** *MYB26* are displayed as the amounts of relative expression compared to those of WT under control conditions. **(G)** A TUNEL assay was performed in WT and P07 lines under both control and drought stress conditions. A TUNEL signal can be seen in the tapetum cells; autofluorescence/TUNEL signal was observed in the pollen grains. V, vascular bundle; PG, pollen grains; T, tapetum. Bars represent the means of 5 plants ± SD. Points with ^*^ and ^**^ differ significantly from the WT (*P* < 0.1 and *P* < 0.05, respectively). Scale bars = 150 μm.

Pollen development demands high levels of energy, which is provided by the mitochondrial metabolism in the tapetum cells to provide nutrients for the increased rate of cell division to produce pollen grains (Pacini et al., 1985). This process is accompanied by a 20-fold increase in mitochondrial numbers compared to that of vegetative tissues (Lee and Warmke, 1979). By boosting mitochondrial biogenesis (Barreto et al., 2014), the UCP1-oe lines sustain high metabolic activity during drought stress that is accompanied, due to increased uncoupled respiration, by decreased production of ROS. Therefore, we determined whether the expression of the transcription factors DYSFUNCTIONAL TAPETUM 1 (DYT1) and DEFECTIVE IN TAPETUM DEVELOPMENT AND FUNCTION 1 (TDF1), which are both important regulators of pollen development (Wilson et al., 2011), were altered in UCP1-oe. These TFs are involved in early, intermediary and late tapetum development. Tapetum development is initiated by *DYT1* and *TDF1*, which were both severely inhibited by drought stress (Figures 11A,B). While *DYT1* (Figure 11A) expression was maintained at high levels in UCP1-oe lines under drought stress, no differences were observed for *TDF1* (Figure 11B). Additionally, while *DYT1* expression was 50% higher in UCP1-oe under control conditions, that of *TDF1* was 30% lower. DYT1 interacts with three bHLH TFs (bHLH010, bHLH89, and bHLH91) to regulate the expression of its targets in Arabidopsis (Zhu et al., 2015). The tobacco ortholog of bHLH89 was inhibited under drought stress in WT plants compared with that in UCP1-oe lines (Figure 11C). Interestingly, the expression of bHLH89 under control conditions was also 30% lower in UCP1-oe lines than in WT plants. These early regulators coordinate the expression of *AMS* and *MS1*, which are involved in the maturation of the tapetum cells, pollen wall formation, and tapetum programmed cell death (PCD). During drought stress, *AMS* expression was 50% lower in WT but was practically unaltered in UCP1-oe lines (Figure 11D). The *MS1* expression pattern changed in the opposite direction; *MS1* expression after drought stress was 37-fold higher in WT and more than 70-fold higher in UCP1-oe lines (Figure 11E). During recovery, *MS1* expression was maintained at high levels in the UCP1-oe lines compared to the levels of WT. After tapetum PCD, there is a final stage of dehiscence during which the TFs that regulate the endothecium and secondary thickening are expressed (Wilson et al., 2011). MYB26, which is expressed at this later stage, was also similarly inhibited under drought stress between WT and UCP1-oe lines (Figure 11F). Interestingly, MYB26 expression was positively affected in the UCP1-oe lines in control conditions.

To verify if the impact of UCP1-oe on the expression of pollen-specific TFs would result in tapetum PCD, we performed a TUNEL assay on the anthers of WT and UCP1-oe line P07 under control and drought stress conditions (Figure 11G). There was no staining of the WT tapetum and P07 anthers under control conditions, but after 15 days of IW, tapetum PCD was observed via the consistent staining of the entire tapetum layer that surrounds the anther (Figure 11G).

### UCP1-oe increases yield and seed size of tobacco plants under drought stress

**Figure 12:**
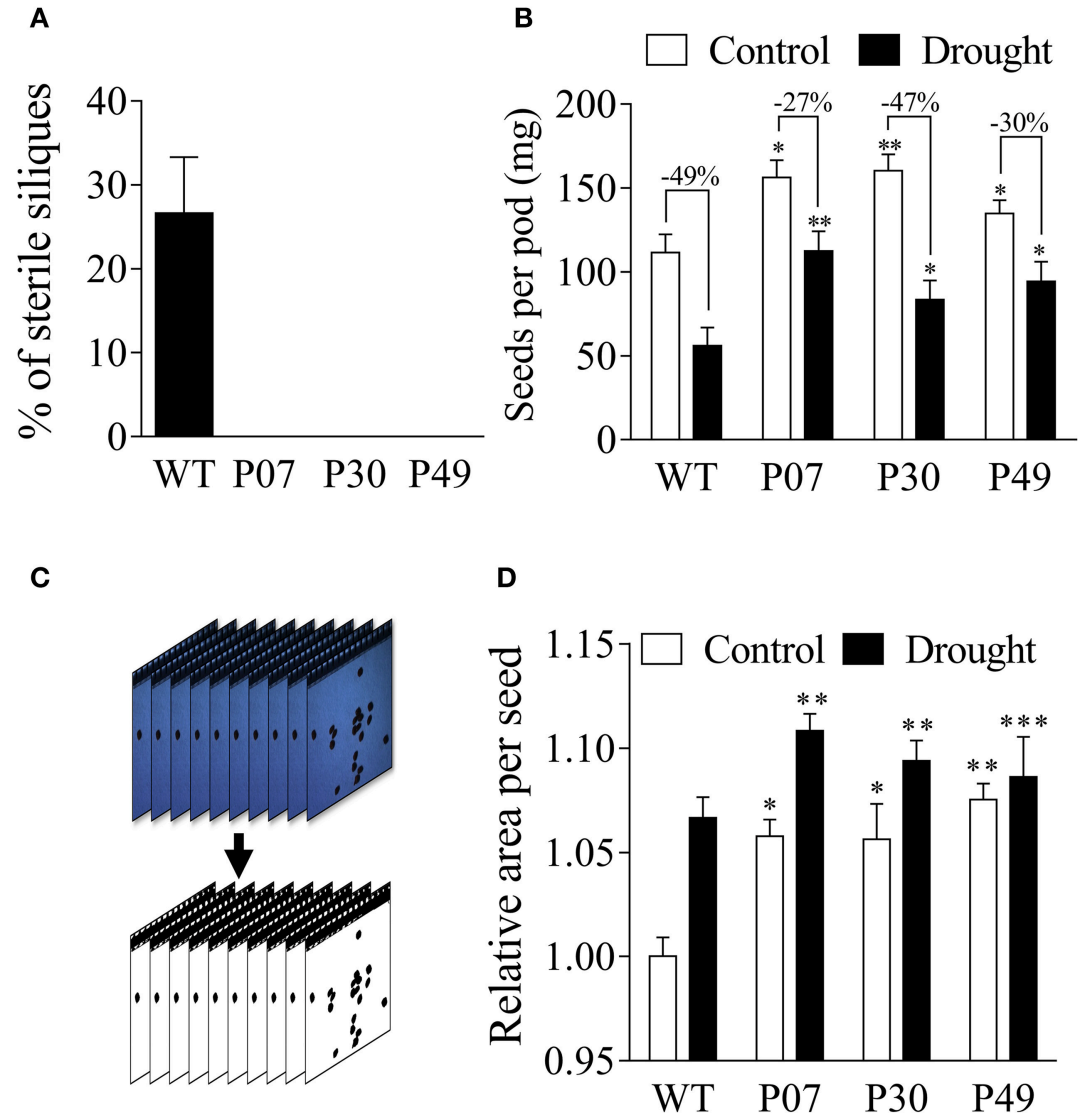
Estimation of the yield of WT and transgenic lines under drought stress. Flower buds (8–12 mm) from control and drought-stressed plants (WT and transgenic) were tagged and maintained under normal irrigation until the end of plant life cycle. **(A)** Transgenic plants did not produce any sterile pods after being subjected to a 15-day drought stress, whereas 25% of WT siliques were sterile. **(B)** WT plants produced fewer seeds under control conditions and were more affected by drought stress compared to transgenic lines. **(C)** Seeds were collected from the pods as described in the Materials and Methods, and a field image containing approximately 100 seeds was collected for each pod. We removed the background and adjusted the contrast to measure the individual seed size using ImageJ. **(D)** Transgenic plants presented larger seeds under control conditions than did WT plants. The seed size increased equally by approximately 5% in all genotypes after drought treatment. Bars represent the means of 25 pods and 2,500 seeds ± SD for seed mass and area, respectively. Bars with ^*^, ^**^, and ^***^ differ significantly from the WT (*P* < 0.1, *P* < 0.05, and *P* < 0.01, respectively).

The impact of UCP1-oe on seed yield under drought stress was evaluated (Figure 12). WT plants that were subjected to drought stress presented 25% sterile siliques, whereas none of the UCP1-oe siliques were sterile (Figure 12A). The mass of seeds per silique increased in the control and drought-stressed UCP1-oe lines compared to that of WT (Figure 12B).

To determine if the increased yield resulted from the increase in seed number or mass, the individual seed area for WT and UCP1-oe lines was analyzed (Figures 12C,D). Images of seeds were taken, and the image backgrounds were filtered to compute the area of individual seeds (Figure 12C). The UCP1-oe lines presented 5% increase in seed area as compared to the WT under control conditions (Figure 12D). Under drought stress, both WT and transgenic lines showed increased seed size compared to that in control conditions; either way, UCP1-oe still presented 5% higher seed area than did WT (Figure 12B). Despite the increase in seed area, most of the difference in yield is due to an increase in seed number (40% increase in silique mass) rather than an increase in seed size (5% increase in seed area).

## Discussion

Although, the mechanistic effects of UCP1-oe are not yet clearly understood (Barreto et al., 2016), it has been observed that UCP1-oe confers tolerance to multiple stresses (Brandalise et al., 2003; Smith et al., 2004; Begcy et al., 2011; Chen et al., 2013; Barreto et al., 2014). Nevertheless, all these studies thus far have been conducted on plants during the vegetative phase of development. Here, we assessed the performance of UCP1-oe lines under drought stress during flower development as well as seed yield under water deficit conditions. The data obtained support the notion that UCP1-oe during the reproductive phase of development allows sustained cellular metabolism and counteracts H_2_O_2_ production in leaves and flowers under moderate drought stress. UCP1-oe produces a less toxic cellular environment in anthers, which contributes to maintaining proper pollen development and seed production.

UCP1-oe lines were previously evaluated under an artificial drought stress simulated by watering plants with a hyperosmotic mannitol solution (Begcy et al., 2011). The use of this setup offers experimental advantages, such as the control of both the environment and the stress level as well as low variability, although it is inherently imperfect at mimicking the drought stress conditions plants are exposed to in the field (Verslues et al., 2006; Lawlor, 2013; Claeys et al., 2014; Porter et al., 2015). In this work, we grew plants in an environment that was more similar to field conditions to evaluate the reproductive phase of development, especially seed production. We could observe for the first time that our transgenic lines present high UCP1 expression and protein content in flower buds (Figures 1G,H). Also, UCP1 protein accumulates in WT under drought stress, which reinforces the importance of UCP1 for stress tolerance.

The transport of photoassimilates during plant development depends on source supply and sink demand (Minchin et al., 2002; Lemoine et al., 2013). Thus, the inhibition of photosynthesis by drought stress is extremely harmful, as the leaf supply of energy for flower and seed development is disturbed. We observed that UCP1-oe does not reduce water losses compared to WT under drought stress, which suggests that the beneficial effect on yield is not a direct result of plant moisture content. The drought stress imposed by withholding water in this work was considered moderate based on the RWC reduction to 65% (Dahal et al., 2014, 2015) and the fact that V_cmax_ is similar between control and recovered plants (Supplementary Table 3). At first, we intended to impose severe drought stress to evaluate the UCP1 effect when the RWC drops below 60%, but we observed that lower RWCs resulted in many aborted flower buds, which would interfere with our analysis. Although, the RWC did not change between WT and transgenic plants, UCP1-oe lines recovered faster than the WT plants after rewatering and presented a decreased loss of biomass. Drought stress is often associated with a reduction in the biomass of aboveground organs in monocots (Sivamani et al., 2000; Wang et al., 2005) and dicots (Sánchez-Rodríguez et al., 2010; Begcy et al., 2011). In this scenario, the beneficial effects of UCP1-oe could be attributed to the preservation of physiological parameters and decreased H_2_O_2_ production. We previously hypothesized that the increase in A_N_ might be a consequence of stomatal function (Barreto et al., 2016) and, in fact, there is a high correlation between G_S_ and A_N_ in both WT and UCP1-oe lines (Figure 3C). Nevertheless, the intercepts of the transgenic A_N_ × G_S_ curves were increased (Figure 3A and Supplementary Table 2). In theory, this means that when there is no gas exchange (G_S_ = 0) there is an increase in photosynthesis in UCP1-oe plants, and consequently, G_S_ is not the only responsible for the increased A_N_ (Gago et al., 2016). In the same direction, G_S_ is inhibited by CO_2_ in a similar manner in WT and UCP1-oe under control and recovery conditions. We hypothesize that the increased mitochondrial respiration, by stimulating TCA cycle, provides CO_2_ for the chloroplast and increases photosynthesis independently of G_S_. Nevertheless, the maintenance of A_N_ in the UCP1-oe plants under drought could be at least partially attributed to the maintenance of G_S_ since it is less sensitive to the environment under drought stress (Figure 4B). We could also observe from the data obtained from the CO_2_ response curves that V_cmax_ did not differ between genotypes under control conditions, but there is a difference in A_N_ under high CO_2_ concentration where carboxylation is limited by Ribulose 1,5-bisphosphate (RuBP) regeneration (Sharkey et al., 2007; Figure 4 and Supplementary Table 3). Under this condition A_N_ is limited by the availability of inorganic phosphate released during the synthesis of sucrose and starch (Sharkey et al., 2007; Flexas et al., 2012; Gago et al., 2016). We have previously shown that UCP1-oe lines accumulate sucrose and starch in the leaves under control conditions (Barreto et al., 2016), therefore it could be hypothesized that there is a decreased impact on A_N_ under RuBP-limiting conditions in these plants.

It has been shown that G_S_, G_M_, and RuBP regeneration have a direct impact on photosynthesis (Gago et al., 2016). Nevertheless, the only direct impact of UCP1 overexpression is on respiration (Vercesi et al., 2006). It is then counterintuitive that a process that contributes negatively to A_N_ would, in a global picture, result in an increased A_N_. It is important to point out that high UCP1 activity should result in high uncoupled respiration (high oxygen consumption) but low ATP production. This means that it is not only respiration that is increased but the pattern of respiration is modified (coupled to uncoupled). We observe that a hypoxic response is triggered in these conditions, which leads to an accumulation of metabolites in the leaves that are markers of hypoxic adaptation such as glucose, sucrose, starch, fumarate, and succinate (Barreto et al., 2016). These metabolites are also markers of photosynthetic activity (Gago et al., 2016), suggesting a strong interplay between hypoxia, carbon fixation, and mitochondrial metabolism. It is known that down regulation of succinate dehydrogenase in tomato and Arabidopsis results in the accumulation of succinate and enhancement of photosynthesis (Araujo et al., 2011; Fuentes et al., 2011; Gago et al., 2016). The increased synthesis of succinate in Arabidopsis is also strongly correlated with A_N_ and G_S_ (Ishihara et al., 2015). In this context, the accumulation of succinate in the UCP1-oe leaves due to increased mitochondrial respiration and TCA activity play a role in the signaling for increased photosynthetic capacity. Nevertheless, sucrose, glucose, and succinate decreased in flower buds, suggesting an inverse correlation between the amounts of these metabolites in leaves and flower buds.

Also, it was found a strong correlation between the maintenance of respiration during drought, and enhanced photosynthetic performance in plants overexpressing AOX has been observed (Dahal et al., 2014, 2015). The inhibition of both UCPs and AOX has a direct effect on photosynthetic performance. The inhibition of AOX decreases photosynthesis in wheat plants suffering from drought stress (Bartoli et al., 2005; Vanlerberghe, 2013), and mutants lacking Arabidopsis UCP1 showed reduced photosynthetic performance under control conditions (Sweetlove et al., 2006). Drought stress induces both UCP1 and AOXs in Durum wheat (Pastore et al., 2007); therefore, the beneficial effects of UCP1-oe when plants are subjected to drought might be similar to those observed for AOX-oe in tobacco (Dahal et al., 2014, 2015). Based on our observation that UCP1 protein content increases under drought stress in WT plants, we can infer that UCPs might have a role in counteracting drought in tobacco flower buds. These observations suggest there could be a connection between UCP1 and AOX function in plants.

The expressions of these proteins are at least partially correlated when either UCP1 is knocked out (Sweetlove et al., 2006) or UCP1 is overexpressed (Barreto et al., 2014). It seems that both proteins have the same function since they ultimately reduce ATP yield (Borecký et al., 2006). We believe it is more appropriate to say that both the UCP1 and AOX proteins lower the amount of O_2_ and substrate consumed per molecule of ATP generated (i.e., respiration efficiency). A more in-depth look at how both proteins influence this reduction in efficiency reveals how different they are and how they could complement each other. Increased AOX activity will decrease both the ratio of NADH consumption and generation of the proton gradient, indicating that AOX would have to consume more substrate to produce the same amount of proton gradient because AOX bypasses proton-pumping complexes III and IV (Vanlerberghe, 2013). The fundamental fact is that AOX does not directly influence the dissipation of the proton gradient—only its production. On the other hand, an increase in UCP activity will consume the proton gradient without directly affecting the efficiency in the consumption of reducing power (Vercesi et al., 2006). Thus, only together can these proteins result in less efficient respiration both in the supply (substrate consumption per proton pumped) and the demand (proton dissipation per ATP yield) parts of mitochondrial respiration. In a situation where there is excess reducing power because photosystems continue generating NADPH and mitochondrial respiration would be adenylate restricted, such as that which can occur during drought stress, the activity of both proteins would complement each other.

The other side of the plant energy balance relies on sink demand for energy, which is known as sink strength or sink dominance (Lemoine et al., 2013). The basis of sink strength is the ability to lower the concentration of photoassimilates in the sinks to establish a favorable hydrostatic pressure gradient between the source and the sink (Wardlaw, 1990). We observed in this study a decreased concentration of sugars and increased expression of mitochondrial genes in flower buds of UCP1-oe lines compared to WT plants. In contrast, we previously reported increased carbohydrate contents in transgenic leaves (Barreto et al., 2016). Drought stress is often associated with increased concentrations of sugars in flower pods and further reductions in yield (Setter et al., 2001; Andersen et al., 2002; Liu et al., 2004), which suggest that the utilization of flower resources rather than sugar concentration is the limiting factor for yield (Liu et al., 2004). These data in conjunction with a higher respiration rate of isolated mitochondria (Barreto et al., 2014) may result in an increased sink strength of UCP1-oe flowers both under control and drought stress conditions, which would be sustained by enhanced photosynthesis and increased carbohydrate content in the leaves. Our data suggest that while in source tissues there is a positive correlation of carbohydrate content and A_N_, in sink tissues this correlation is negative (Figure 10C). Apparently, this is not true for succinate, which is positively correlated with A_N_ in both leaves (Sivamani et al., 2000; Barreto et al., 2016) and flower buds (Figure 10C). The capacity of source tissues to provide energy for a strengthened sink resulted in less accumulation of H_2_O_2_ in UCP1-oe lines. We also observed that compatible osmolytes that are broadly associated with increased drought tolerance, such as proline, did not differentially accumulate between WT and UCP1-oe plants.

The impact of drought stress on tapetum development, which is a high energy-demanding process in plants for sustaining pollen formation, was analyzed by profiling the expression of DYT1, TDF1, bHLH89, AMS, MS1, and MYB26. We found that DYT1, bHLH89, AMS, and MS1 were less inhibited under drought stress in UCP1-oe lines than in WT plants. Some of these TFs were affected under control conditions, which indicates that UCP1-oe impacts tapetum metabolism. There were no apparent differences in tapetum PCD between UCP1-oe and WT plants when drought stress conditions were compared to control conditions. Thus, it is not clear how UCP1-oe affects the expression of these TFs and what the consequence of the downstream processes are during tapetum development.

The positive effect of UCP1-oe on leaf photosynthesis and flower bud metabolism resulted in a higher yield compared to that of WT under both control and drought stress conditions. Drought stress affects reproductive tissue development by affecting pollen grain viability, ovary development, and turgor maintenance of floral organs (Alqudah et al., 2011). This study focused on the early stages of anther development because of the importance of mitochondria in this process. Since pollen development depends on a high energy supply, disturbances in the function of mitochondria might result in dramatic effects on male fertility (Chen and Liu, 2014). Mitochondria play a fundamental role in triggering the death of male reproductive organs (Chase, 2007), not only by affecting the levels of ATP (Li et al., 2013) but also by their involvement in increased ROS production (Luo et al., 2013). In this context, we believe that the positive effects observed on yield are because UCP1-oe can maintain mitochondrial respiration and photosynthesis in source leaves independent of the consequences of water limitation compared with WT plants; UCP1-oe, therefore, does not limit the ATP requirement of reproductive tissues, avoiding ROS production.

Nevertheless, from the observations made in this study, it is important to note that the effect of UCP1 was not drought specific because it also improved plants in well-watered conditions. At this point, it's hard to establish the basis of the resistance of UCP1-oe plants to biotic and abiotic stresses. UCP1 activity *per se* is beneficial to mitochondrial metabolism itself as well as to the whole cell because it reduces ROS production by stimulating respiration when needed (Borecký et al., 2006; Vercesi et al., 2006). We believe that this fact alone contributes to the phenotype, but we are not able to conclude its extent, mainly because there are some side effects of constitutive UCP1-oe (Barreto et al., 2014, 2016). The first is the fact that mitochondrial biogenesis is induced in transgenic lines (Barreto et al., 2014). The expression of multiple mitochondrial components such as AOX, SOD, catalases, and Krebs cycle enzymes (Barreto et al., 2014) amplifies the UCP1 effect by improving the whole mitochondrial antioxidant activity, steps of the Krebs cycle and oxidative phosphorylation. We also cannot determine the share of the mitochondrial biogenesis effect on the UCP1 phenotype. Also, there is an interesting secondary side effect of a hypoxic response that is triggered in these plants (Barreto et al., 2016). We found that when UCP1 is overexpressed, it triggers a hypoxic response that induces components of the N-end rule pathway of the hypoxic response, leading to accumulation of specific metabolites that are markers of the hypoxic response (Barreto et al., 2016). Beneficial effects of the activation of the N-end rule pathway upon stress tolerance constitute a widespread and a trending topic in plant sciences (Gibbs et al., 2011); therefore, we also cannot conclude how the N-end rule pathway contributes to the UCP1 phenotype. The evaluation of how each of these effects contributes to stress tolerance and of the interplay between UCP1 and AOX would have a significant impact on the understanding of how plants manage metabolic imbalances and might be a valuable tool to improve crops for increased yield under stresses.

## Author contributions

PB, JY, ZW, and PA designed the experiments. PB and JY performed experiments. PB, JY, ZW, and PA analyzed the data. PB and PA wrote the paper.

### Conflict of interest statement

The authors declare that the research was conducted in the absence of any commercial or financial relationships that could be construed as a potential conflict of interest.

## Supplementary material

Supplementary Figure 1 Experimental design for assessment of the impact of drought stress in UCP1-oe tobacco plants. **(A)** WT and UCP1-oe lines (P07, P30, and P49) were grown in 3 different groups (control, drought and recovery) containing 20 plants each (5 per genotype). **(B)** Plants were grown for 12 weeks until they reached the reproductive phase of development. Samples were collected from the control group **(C)** before we stopped irrigation (*T* = 0). Drought **(D)** samples were collected after 15 days of withholding water (*T* = 15), after which irrigation was resumed. Plants were allowed to recover under normal irrigation for 24 h, after which the recovery (R) samples were collected (*T* = 16). Plants from all groups were maintained until harvest.

Supplementary Figure 2 Effects of drought stress on physiological and chlorophyll fluorescence parameters of WT and *AtUCP1*-overexpressing transgenic plants on the second trial of the experiment. The measurements were taken at growth light intensity (400 μmol m^−2^ s^−1^) using the first expanded leaf during the whole course of the experiment. For this experiment, plants were allowed to recover from drought stress for 3 days. **(A)** Respiration in the dark: (R_D_), **(B)** respiration in the light (R_L_), **(C)** net photosynthetic rate (A_N_), and **(D)** stomatal conductance (G_S_).
