# Supplementary figures and images for: Mitochondrial Uncoupling Protein 1 Overexpression Increases Yield in Nicotiana tabacum under Drought Stress by Improving Source and Sink Metabolism
Source: Front Plant Sci. 2017 Nov 1;8:1836. doi: 10.3389/fpls.2017.01836 (PMC5672497; doi:10.3389/fpls.2017.01836)

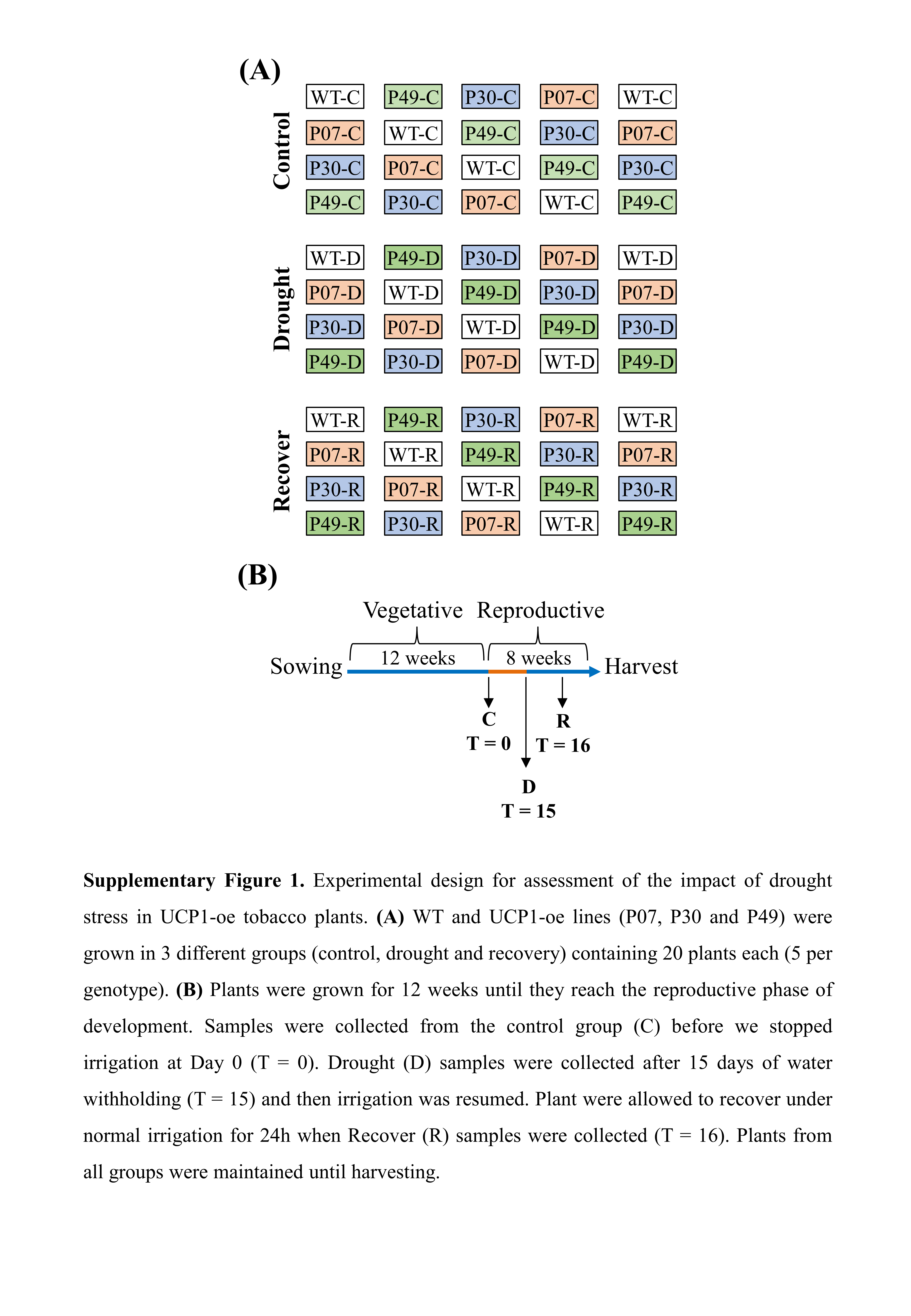

Supplement: Supplementary Figure 1 — Experimental design for assessment of the impact of drought stress in UCP1-oe tobacco plants. (A) WT and UCP1-oe lines (P07, P30, and P49) were grown in 3 different groups (control, drought and recovery) containing 20 plants each (5 per genotype). (B) Plants were grown for 12 weeks until they reached the reproductive phase of development. Samples were collected from the control group (C) before we stopped irrigation (T = 0). Drought (D) samples were collected after 15 days of withholding water (T = 15), after which irrigation was resumed. Plants were allowed to recover under normal irrigation for 24 h, after which the recovery (R) samples were collected (T = 16). Plants from all groups were maintained until harvest. [file Image1.TIF]

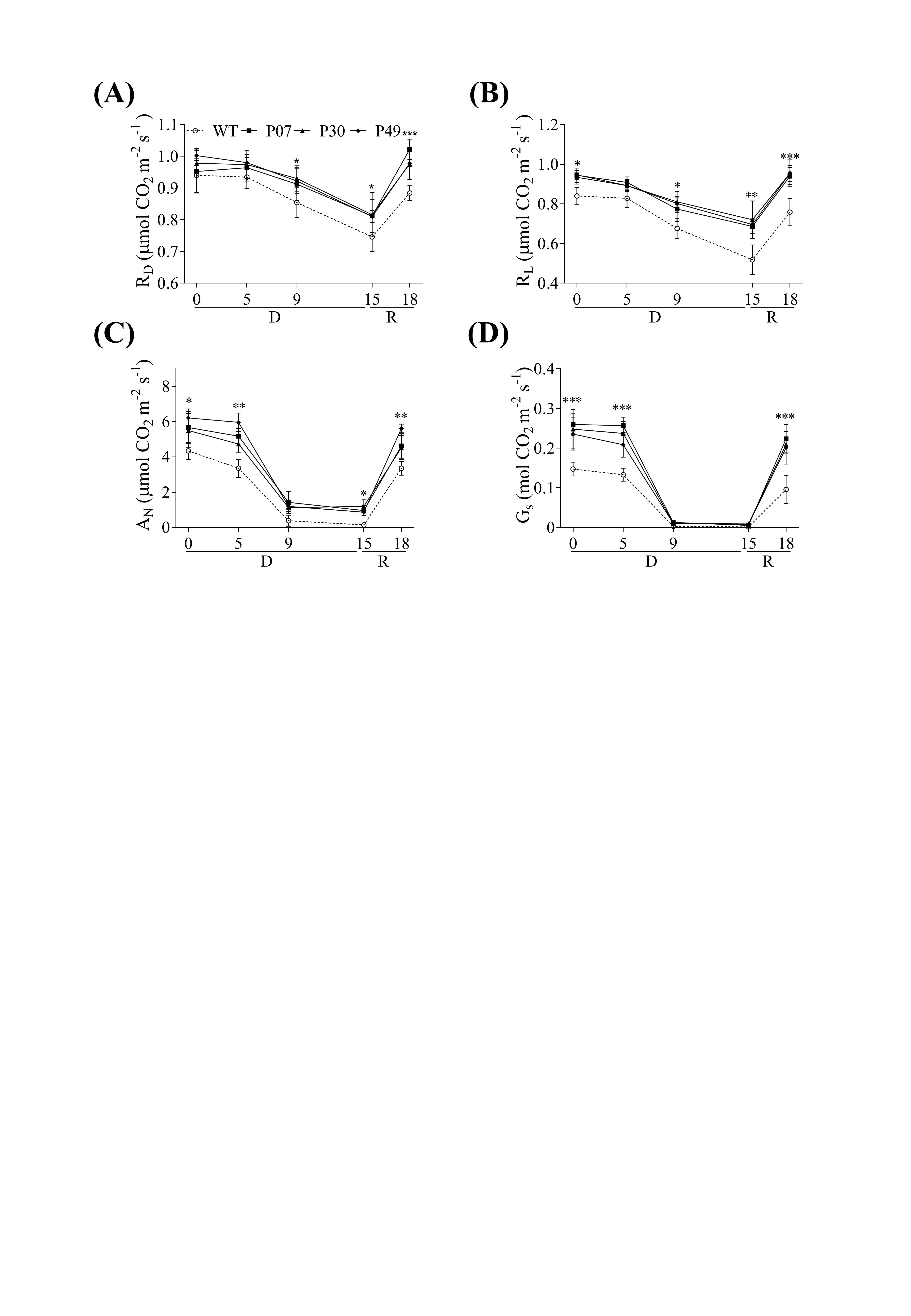

Supplement: Supplementary Figure 2 — Effects of drought stress on physiological and chlorophyll fluorescence parameters of WT and AtUCP1-overexpressing transgenic plants on the second trial of the experiment. The measurements were taken at growth light intensity (400 μmol m−2 s−1) using the first expanded leaf during the whole course of the experiment. For this experiment, plants were allowed to recover from drought stress for 3 days. (A) Respiration in the dark: (RD), (B) respiration in the light (RL), (C) net photosynthetic rate (AN), and (D) stomatal conductance (GS). [file Image2.TIF]
